# Inferring mobility reductions from COVID-19 disease spread along the urban-rural gradient

**DOI:** 10.3389/fpubh.2026.1840916

**Published:** 2026-07-06

**Authors:** Sydney Paltra, Jonas Dehning, Viola Priesemann, Kai Nagel

**Affiliations:** 1 Technische Universität Berlin, FG Verkehrssystemplanung und Verkehrstelematik, Berlin, Germany; 2 Max Planck Institute for Dynamics and Self-Organization, Göttingen, Germany; 3 Faculty of Physics, Institute for the Dynamics of Complex Systems, Georg-August-University Göttingen, Göttingen, Germany

**Keywords:** Bayesian inference, behavior adaptation, COVID-19, mobility data, mobility reduction, structural equation modeling, socioeconomic factors

## Abstract

The COVID-19 pandemic reshaped human mobility through interventions and voluntary behavioral changes. Those mobility reductions helped mitigate disease spread, but factors driving variation in mobility reduction remain unclear. We introduce a Bayesian hierarchical model to quantify heterogeneity in mobility responses across time and space in Germany's 400 districts using anonymized phone data. The model successfully reproduces timing and magnitude of major reductions in mobility across districts, revealing that disease spread affected mobility reductions most strongly [effect during first wave: −23%, IQR: (−27%, −20%), second wave: −18% (−21%,−15%)], followed by temperature (median difference of 11% between summer and winter), school vacations (−4%), and public holidays (−3%). We find significant differences in mobility response along the urban-rural gradient, with large cities reducing mobility most strongly. Investigating socioeconomic influences on reaction strength reveals different patterns across waves: during the first wave, mainly population density and employment variables are significant predictors (adj. *R*^2^ = 0.44), while population density and political variables mainly explain the variance during the second wave (adj. *R*^2^ = 0.37). Our results highlight that mobility serves as a valuable behavioral proxy with near real-time availability, making it an essential information source for future outbreaks.

## 1 Introduction

During the COVID-19 pandemic, people substantially adapted their mobility patterns as case numbers increased and official guidelines and policy evolved: social visits were postponed, meetings and shopping shifted online, public transport during peak-hour was avoided, stay-at-home rules were observed, and school were closed. These adjustments combined both voluntary adaptations due to increased personal risk and compliance to government-mandated non-pharmaceutical interventions (NPIs), making mobility an immediate indicator of population response (1–3). Indeed, investigations show that time spent away from home mediates exposure opportunities; fewer minutes spent in shared spaces translate into fewer contacts and a lower effective reproduction number (1, 4). Because many control measures directly affect mobility, tracking it shows whether interventions take effect, where additional support is needed, and how quickly behavior adapts. Importantly, these responses can be measured in near real time, due to the availability of anonymized mobile-phone data at population scale (5, 6).

The magnitude and timing of mobility reductions differ across time and space. Voluntary responses and NPI compliance depend on local information environments and social norms, workplace constraints and the feasibility of remote work, and, critically, trust in institutions (7–9). Furthermore, structural conditions, including housing density, income, work opportunities, infrastructure, and dependency on public transport, differ along the urban-rural gradient, so the same NPIs and case numbers can yield different reductions in out-of-home duration in different locations. This variation matters: it changes when and where transmission accelerates, and it determines whether finite resources such as testing and hospital capacity are deployed equitably and most effectively. Yet most studies quantify mobility adaptations at national or international scales or, when subnational, focus on metropolitan areas (10, 11), leaving rural and small-town contexts comparatively underexplored (12, 13). Improving the understanding of how responses vary across the urban-rural gradient fills an essential research gap that will improve transmission modeling, targeted communication, and fair allocation of interventions (14, 15).

To quantify differences along the urban-rural gradient, we focus on Germany during the first year of the COVID-19 pandemic, quantifying changes in local mobility for all 400 German districts (*Landkreise* and *kreisfreie Städte*). We use out-of-home duration, the amount of time spent away from one's home, as our mobility measure, drawing on anonymized cellular data from a major German mobile telecommunications provider. This sample encompasses up to 30 million devices (6, 16). Unlike reported case incidence, which serves as a key epidemiological metric but represents a delayed and composite behavioral signal influenced by testing, reporting practices, baseline transmissibility, and other protective behaviors, out-of-home duration offers a more immediate and direct measure of behavioral response to COVID-19.

In this study, we develop a Bayesian hierarchical model that decomposes out-of-home duration into a disease-responsive component and three disease-independent drivers (temperature, school vacations, and public holidays). The disease term combines national and local incidence through a weighted finite-memory signal and allows for pandemic fatigue, yielding a district-specific reaction strength to disease spread. Our model successfully disentangles these components, finding that disease is the leading factor impacting out-of-home duration (median reduction first wave: 23%, second wave: 18%), followed by temperature (median seasonal effects: 11%), school vacations (4%), and public holidays (3%). Local incidence carries only a minority weight compared to national incidence in explaining mobility adaptations, though we observe a partially significant trend of increasing local weight from urban to rural districts. An ANOVA test reveals significant (*p* < 0.0001) differences in mean reaction strength across district types, while subsequent pairwise *t*-tests reveal significant differences (*p* < 0.001) along the urban-rural gradient, with large cities reducing most strongly. Investigating demographic and socioeconomic influences, we find that urban-rural differences, together with the social and economic variables, explain the variance in reaction strength across districts well (adj. *R*^2^ first wave: 0.44, second wave: 0.37). However, these differences in reaction strength only partly translate into a stronger reductions in peak incidence. Overall, our work deepens the understanding of pandemic-mobility interrelations. The results highlight that mobility data, which is available in near real-time, can serve as a valuable behavioral proxy during pandemic response, supporting decision makers and researchers alike.

## 2 Results

### 2.1 Analysis overview

COVID-19 shaped the 2020 annual course of the out-of-home duration in Germany's 400 districts through a combination of government-mandated interventions and voluntary cutbacks in social and work related travel (3, 17). Our objective is to quantify the strength of this joint effect and to identify the demographic and socioeconomic characteristics that influenced it. Because time spent outside the home mediates exposure opportunities, reductions in out-of-home duration strongly affect transmission dynamics and thus a wave's incidence peak (4). At the same time, peak incidence reflects additional influences, including protective behaviors like mask-wearing or vaccinations and differences in the baseline reproduction numbers across districts. Consequently, we regress both mobility reduction and peak incidence on socioeconomic and demographic factors to understand their cross-dependencies. We structure the analysis in three steps:

1. A Bayesian model for weekly decreases in out-of-home duration driven by disease spread alongside three disease-independent factors,
2. A linear model attributing cross-district variation in these decreases to population density and demographic and socioeconomic covariates,
3. A structural equation model attributing cross-district variation in peak incidence to the same covariates and additionally to the average decrease in out-of-home duration.

### 2.2 Impact of disease spread on out-of-home duration

**Figure 1:**
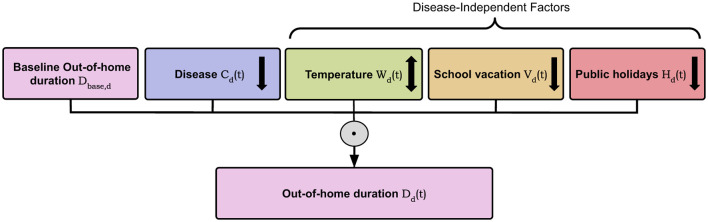
Model overview illustrating the composition of the time- and district-dependent out-of-home duration. The out-of-home duration is the product of a constant baseline out-of-home duration, a disease factor (based on incidence) and three disease-independent factors (temperature, school vacations, public holidays). Arrows indicate if we assume the factor to have an in- and/or decreasing effect on the out-of-home duration.

Our Bayesian model spans the 52-week period from March 2020 to March 2021. The multiplicative model represent the out-of-home duration *D*_*d*_(*t*) for each week *t*∈*T* = {1, …, 52} and each German district *d*∈*D* = {1, …, 400}. We assume that the out-of-home duration is determined by a baseline out-of-home duration, COVID-19 disease spread, and by three disease-independent factors: temperature, school vacations, and public holidays (see Figure 1 for a reduced graphical representation of the model and Table 1 for introduction of underlying data bases and sources).

**Table 1:** Data bases for the dependent variable *D*_*d*_(*t*) and the inferred factors in the Bayesian analysis.

Factor	Based on	Source
*D*_*d*_(*t*)	Cell-based mobility data (dependent variable). We define out-of-home duration as the daily time (in hours) individuals spend outside the home cell. It was aggregated across all postal codes within each district and averaged over all recorded individuals. We use weekly averages (see (16) for details on aggregation and cell-based mobility data)	Balmer et al. (52)
*C*_*d*_(*t*)	COVID-19 7-Day Incidence rate for each district *d* and national COVID-19 7-Day Incidence rate	RKI (53)
*W*_*d*_(*t*)	Weekly average of daily maximum temperature (in °C) for district *d*. If daily maximum temperature was unavailable any time during the study period for district *d*, we considered the average daily maximum temperature of the neighboring districts	Meteostat (54)
*V*_*d*_(*t*)	School vacations. They differ on a federal state level, not district level. Sum of school vacation days for week *t*. Children in Germany go to school Monday–Friday; maximum number of school vacation days in week *t* is 5	Schulferien.org (55)
*H*_*d*_(*t*)	Public holidays. They differ on a federal state level, not district level. Sum of public holidays that fall on weekday in week *t*	Schulferien.org (55)

Exploratory analysis of input variables may be found in Section 4.1, while the exploratory analysis of the output variable out-of-home duration may be found in Section 4.2.

We assume these factors are multiplicative (i.e., additive in log-space) because we assume changes in one factor do not induce changes in another, and because their effects can be expressed as multiples of a base level. The model reads:


$$\begin{array}{l} D_{d}\left(t\right)=D_{\text{base},d}\cdot C_{d}\left(t\right)\cdot \left(W_{d}\left(t\right)\cdot V_{d}\left(t\right)\cdot H_{d}\left(t\right)\right). \end{array}$$

**Figure 2:**
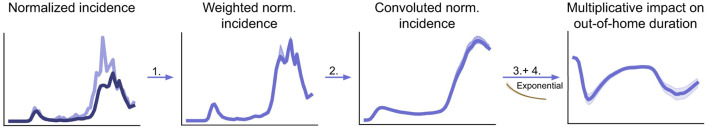
Normalized local and national COVID-19 incidence are used to compute the disease factor in a four step process. 1. Weighted average of normalized local and national incidence is computed. 2. Case numbers are convolved with a Gamma distribution. 3. Multiplication of convolved case numbers with exponential decay function to integrate pandemic fatigue. 4. Exponential of convoluted and exponentially decayed case numbers is taken. In the second to fourth panel, lines represent median values, ribbons correspond to 95% credible intervals.

The baseline out-of-home duration *D*_base, *d*_ is a fitted constant per district *d* representing the out-of-home duration during a week without disease spread, without school vacations, no public holidays, and with annual average temperature. This baseline out-of-home duration *D*_base, *d*_ is multiplied by *C*_*d*_(*t*), a factor depending on the COVID-19 disease spread. The disease factor *C*_*d*_(*t*) is based on the COVID-19 incidence rate (new weekly cases per 100,000 inhabitants) and is computed in four steps (Figure 2 for illustration and Subsection 4.3.5 for details). *First*, based on the assumption that both local and national disease dynamics influence mobility behavior, a weighted average of the normalized local and normalized national COVID-19 incidence rate is computed. *Second*, as the population is not only influenced by the current incidence, but also by that of the recent past, the incidence is convolved with a Gamma distribution. *Third*, assuming that the population's perception of risk decreases over time as pandemic fatigue sets in, the convoluted incidence is multiplied by an exponential decay function. Ergo, the same level of incidence reduces the out-of-home duration less at a later point in time. *Fourth*, assuming disease to have a multiplicative effect, we take the negative exponential of the convoluted and exponentially decayed incidence. This also ensures that positive incidence has a multiplicative effect smaller than one.

The disease factor captures the overall effect of COVID-19 incidence on mobility, encompassing both voluntary and mandatory behavioral changes. On the one hand, rising incidence may prompt individuals to reduce travel voluntarily, but may, on the other hand, also trigger government interventions such as lockdowns that curtail mobility by mandate. Because these two pathways are deeply intertwined and difficult to disentangle empirically, our disease factor combines both; a conflation that should be kept in mind when interpreting the results.

The product of the baseline out-of-home duration *D*_base, *d*_ and disease factor *C*_*d*_(*t*) is further multiplied by three disease-independent factors:

- Temperature factor *W*_*d*_(*t*). When temperatures are high, this factor increases the out-of-home duration, while when temperatures are low, this factor decreases the out-of-home duration (18, 19).
- School vacation factor *V*_*d*_(*t*). School vacations decrease the weekly out-of-home duration, as children do not attend school and parents take time off work to look after their children (20).
- Public holiday factor *H*_*d*_(*t*). Most employees are not required to work on public holidays, leading to a decrease in out-of-home duration (20–22).

The strength of each of these factors is allowed to vary between districts, we only assume that the strength is to some degree similar across districts (hierarchical modeling). We focus on the period from March 2020 to March 2021 because COVID-19 vaccines were not yet widely available during this time frame, thus ensuring that vaccinations did not affect the out-of-home duration. We perform Bayesian inference for the parameters of our model using Markov-Chain Monte Carlo (MCMC) sampling (see Section 4.4 for details).

### 2.3 COVID-19 spread was the most important contributor to mobility changes

**Figure 3:**
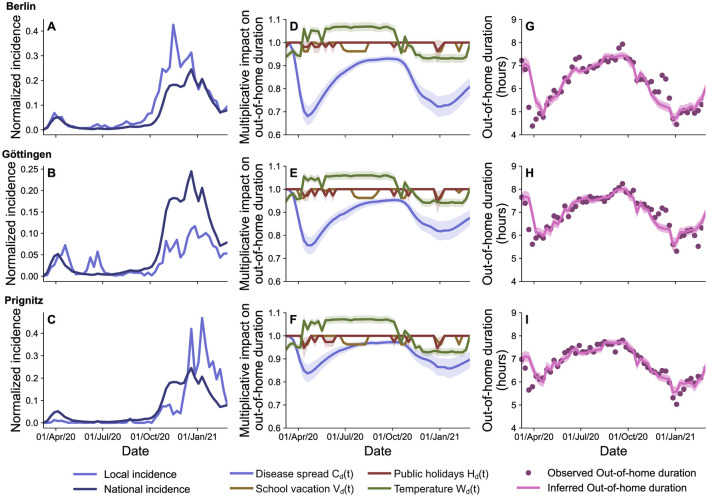
Using Bayesian inference we successfully infer the annual course of the out-of-home duration for the three exemplary districts Berlin, Göttingen, and Prignitz. The authors chose to depict their home districts of Berlin and Göttingen, together with Prignitz, the district with the lowest population density. **(A–C)** Normalized local and national incidences. All three exemplary districts experienced a first (spring 2020) and second (winter 2020/2021) COVID-19 wave. **(D–F)** Multiplicative impact of the disease factor *C*_*d*_(*t*) and the three disease-independent factors *W*_*d*_(*t*), *V*_*d*_(*t*), and *H*_*d*_(*t*). The disease factor *C*_*d*_(*t*) decreases the out-of-home duration most strongly, followed by smaller effects of *W*_*d*_(*t*), and only modest reductions due to *V*_*d*_(*t*) and *H*_*d*_(*t*). **(G–I)** Observed vs. inferred out-of-home duration. The model successfully infers the annual course of the out-of-home duration, specifically the shape of the two major decreases in spring 2020 and winter 2020/2021. In panels **(D–I)**, lines represent median values, ribbons correspond to 95% credible intervals.

Our model successfully fits the observed out-of-home duration between March 2020 and March 2021 (Figure 3 right column for exemplary depictions of Berlin, Göttingen, and Prignitz). ab The model infers the overall shape of the two major decreases in out-of-home duration during the first year of the COVID-19 pandemic: the sharp decline in spring 2020 during the initial COVID-19 wave and the second decline in winter 2020/2021 during the second COVID-19 wave. While the model successfully captures these trends, it leaves opportunity for improvement at two specific time points. First, the model predicts the local minimal out-of-home duration in spring 2020 too late (Model: April 5th vs. March 15th in the data). Second, the model misses a temporary spike in out-of-home duration observed in late November to early December 2020 that interrupted the otherwise declining trend. A potential explanation for this spike is the looming stricter lockdown that was finally announced on December 13th, 2020 and took effect on December 16th, 2020. This encouraged advanced Christmas shopping and increased activity. But overall, the model fits most variance well, including some short-term variations explained by school vacations and public holidays.

In the three exemplary districts, the disease spread had a stronger multiplicative effect on out-of-home duration than the three disease-independent factors (Figure 3 middle column), with substantially larger reductions in spring 2020 than in winter 2020/2021. The three disease-independent factors—school vacations, public holidays, and temperature—significantly influenced out-of-home duration, provide secondary but consistent reinforcing effects, with temperature decreasing mobility until mid-April 2020 and increasing it during the warm summer months (May–September).

**Figure 4:**
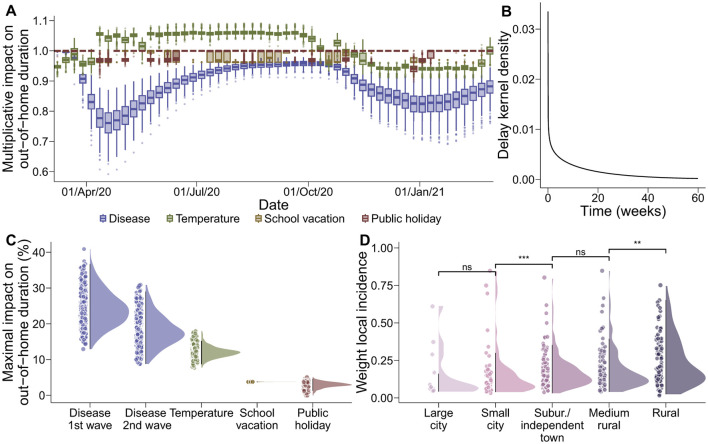
Summary of the Bayesian model's results. **(A)** The influence of the different factors is comparable across districts. Distribution of multiplicative impact across districts, using the inferred median multiplicative impact for each district. Across districts, the disease factor emerges as the strongest influence, with pandemic fatigue evident in every district. Due to temperature fluctuations, out-of-home duration is approximately 10% higher in summer than in winter. School vacations and public holidays lead only to modest reductions. Lines represent median values, boxes represent IQR, whiskers extend from the hinge to at most 1.5*IQR from the hinge, dots are outliers. **(B)** Gamma distribution delays the effect of the weighted normalized incidence. The mean inferred delay between the incidence and observed out-of-home duration is 16 weeks, modeled via a convolution with a gamma kernel. **(C)** The disease factor emerges as the strongest influence on out-of-home duration. Distribution of effect sizes, in other words size of impact on out-of-home duration, across districts, using the median effect size for each district. While the effect size of the disease factor decreases from the first to the second wave, it still influences out-of-home duration most strongly. Secondary effects due to temperature, followed by only modest effects due to school vacation and public holiday. Each dot represents one district. **(D)** Local incidence carries only a minority of the weight in explaining mobility behavior. Distribution of weight of normalized local incidence in disease factor *C*_*d*_(*t*). Across districts, the majority of weight in explaining local mobility behavior is placed on national rather than local incidence, aligning with the introduction of nation-wide NPIs and communication strategies. ANOVA test applied before pairwise comparison finds significant (*p* < 0.01) difference in mean weight across district types. Stars indicate pairwise *t*-test determining difference in mean across district types to be statistically significant, with significance levels of ^***^ representing *p* < 0.001, ^**^ representing *p* < 0.01, and ns representing *p*>0.05.

Expanding the analysis from the three exemplary districts to all 400 German districts (Figure 4A), the disease factor consistently emerges as the strongest influence on out-of-home duration, confirming the pattern observed for Berlin, Göttingen, and Prignitz: During the first wave, the disease factor *C*_*d*_(*t*) reduces the out-of-home duration most strongly in the week ending April 19th, 2020, reaching a median multiplicative impact of 0.77, IQR: [(0.73, 0.80)] (Figure 4C). Despite higher incidences during the winter 2020/2021 wave, the disease factor reduces out-of-home duration less than during spring 2020, reaching a median maximal multiplicative impact of 0.82 [IQR: (0.79, 0.85)]. This suggests that pandemic fatigue fully compensates the effect of higher incidences. The difference in out-of-home duration between winter and summer is about 11%, with a median multiplicative impact of 1.06 [IQR: (1.05, 1.07)] during the warm summer months (May–September) and 0.95 (IQR: [0.94, 0.95]) during the winter. The decreasing impact of school vacations and public holidays is even smaller: The median difference between weeks with school vacations and weeks without is 4% (same effect is inferred for all districts; boxes in Figure 4C represent that during the corresponding week school vacations occurred in some districts but not in others, rather than a difference in effect size). The median difference between weeks with public holiday(s) and without is 3%. To test whether disease spread masks the contributions of temperature, school vacations, and public holidays in the 2020 model, we conducted a sensitivity analysis, fitting the model to 2024 data (Supplementary section 5.2). For 2024, we assume no influence of disease spread on out-of-home duration, but still find effect sizes for temperature, school vacations, and public holidays comparable to those of 2020, suggesting that no such masking occurs in the 2020 model. In summary, across districts, like in the exemplary districts of Berlin, Göttingen, and Prignitz, disease spread most strongly reduces the out-of-home duration, followed by the influence of temperature and modest reductions due to school vacations and public holidays.

Interestingly, the inferred delay kernel between incidence and out-of-home duration is highly skewed. The average delay between incidence and out-of-home duration is 16 weeks, but the median is 10.3 weeks, and the 25th percentile is already reached after 3.6 weeks (the IQR of both the median and the 25th percentile are smaller than one day, Figure 4B). This suggests that a substantial fraction of the behavioral adaptation occurs within approximately 3 weeks, consistent with the expected time frame for responses to media information, perceived epidemic risk and subsequent policy changes. At the same time, a larger part of the response persists for much longer, suggesting an enduring effect even after the immediate epidemic situation has improved.

### 2.4 Stronger mobility reductions in more densely-populated districts

**Figure 5:**
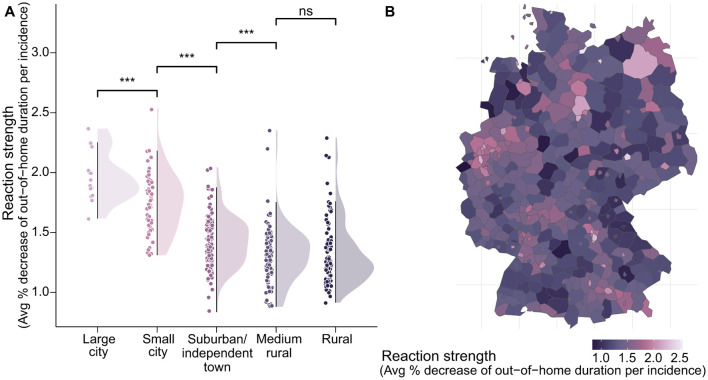
Differentiation by district type and spatial depiction strongest reaction strength in cities and parts of the west of Germany. In either panel, incidence represents new weekly cases per 100,000 inhabitants. **(A)** The ANOVA test applied before the pairwise comparison finds significant (*p* < 0.0001) difference in mean across district types. Pairwise tests reveal that large cities display the greatest average reaction strength, with reaction strength structurally decreasing along the urban-rural gradient. Stars indicate pairwise *t*-test determining difference in mean across district types to be statistically significant, with significance levels of ^***^ representing *p* < 0.001 and ns representing *p*>0.05. **(B)** Depicting the reaction strength spatially highlights great reaction strength in cities (small polygons) and in the west of Germany, contrasting with modest reaction strengths in parts of the former GDR and northern Bavaria.

In order to understand the reasons for the differences in reduction of out-of-home duration between districts, we first investigate how much it depends on rural vs. urban differences. To this end, we classified districts into five district types: large city, small city, suburban/independent town, medium rural, and rural (using the classification system of the *German Federal Institute for Research on Building, Urban Affairs, and Spatial Development*). We did not directly use the reduction of out-of-home duration as our variable of interest, but the *reaction strength* of the out-of-home duration with respect to the incidence. We used reaction strength as we expected that for districts with high local incidence, the decrease in out-of-home duration will be stronger (even if that effect is not so strong, as most of the districts are more strongly tied to the national incidence, Figure 4D). As reaction strength we used the average impact of the incidence on the reduction of out-of-home duration, which is mathematically the integral of our exponential decay function. Comparison across district types revealed large differences: large cities exhibited, on average, the largest reaction strength, while rural districts showed the weakest response (Figure 5A). *T*-tests comparing mean reaction strength across district types confirmed significant differences between the four more densely populated district types: large city vs. small city (*p* < 0.01), small city vs. suburban/independent town (*p* < 0.001), and suburban/independent town vs. medium rural (*p* < 0.001). Finally, the difference in mean reaction strength reaches a plateau, with no statistically significant difference observed between medium rural and rural districts (*p*>0.05). Overall, this demonstrates a clear urban-rural gradient in response to disease spread, with reaction strength decreasing systematically from large cities to rural areas.

However, differentiation by district type does not suffice to fully explain the observed differences in mobility reduction in the 400 districts (see Figure 5B, in general smaller polygons represent cities). For instance, we observe strong reactions in the west of Germany, particularly all across North Rhine-Westphalia. Meanwhile, northern Bavaria (specifically the Upper Palatinate region) shows only moderate reactions, even in the city districts. Furthermore, we are interested in whether urbanness is really the main factor that influences urban-rural differences in mobility reduction, or whether partially-collinear demographic or economic factors also play a role.

**Figure 6:**
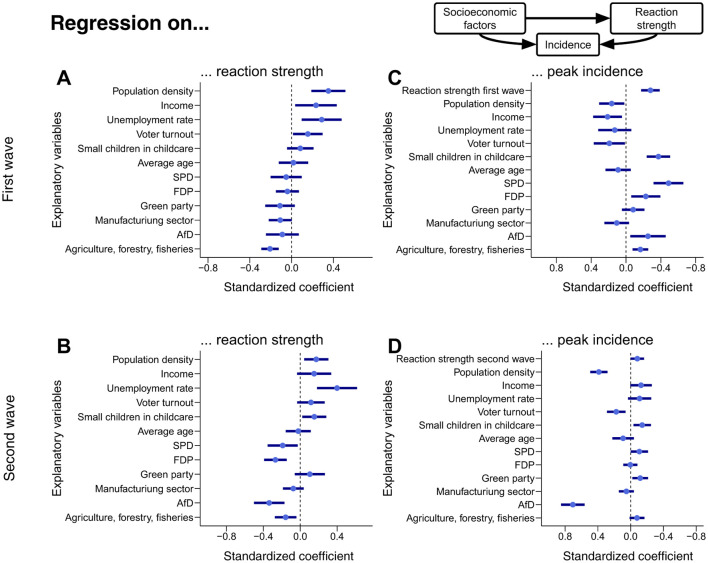
Multiple regressions and structural equation models highlight the complex interrelation of reaction strength and peak incidence. **(A, B)** Regression of demographic, socioeconomic, and political variables on *reaction strength first wave* **(A)** and on *reaction strength second wave* **(B)**, reveals a shift in explanatory factors from economic and demographic factors to political conviction. **(C, D)** Regression of reaction strength and the same demographic, socioeconomic, and political variables on *peak incidence first wave* **(C)** and *peak incidence second wave* **(D)**, highlights that peak height depends, but not exclusively, on mobility reductions. Note that for panels **(C, D)** the *x*-axis has been flipped. In these two panels, negative correlation coefficients have a disease mitigation, i.e., peak incidence decreasing, effect. In all four panels, dots represent standardized coefficients, whiskers correspond to 95% confidence intervals.

To better understand which factors are responsible for the observed differences in mobility reduction, we regressed twenty potentially relevant demographic, socioeconomic, and political variables that encode differences between districts on the *reaction strength during the first wave* and on the *reaction strength during second wave* separately (defined as the integral over the corresponding wave, see Section 4.5 for details). More precisely, as demographic variables we considered population density, average age, and share of 65+ year old people; as economic variables we considered the average income, employment and unemployment rate, economic sectors (agriculture, forestry fisheries, manufacturing sector, construction, service sectors, trace, transport, hospitality, information, communication (TTHIC) sectors, finance sector); and as sociological and political variables we considered the share of small children in childcare, the voter turnout, and share of votes of any of the political parties that received more than 5% of votes in the 2021 federal election (see Supplementary Table S3) for overview and summary statistics). Exhaustive search and comparison of model selection criteria (adjusted *R*^2^, Mallow's *C*_*p*_, AIC, and BIC) determined which explanatory variables were considered in the final regression on reaction strength. In the regression, we then used the set of variables for both waves that are significant during either wave (Figures 6A, B, see also Subsections 4.5.2 and 4.5.3 for details).

We find that during the first COVID-19 wave, *population density* and *unemployment rate* contribute most strongly to explaining variance in reaction strength, with similar strong effects (Figure 6A and Table 2 for detailed model output as well as Table 3 for discussion of potential multicollinearity). The strong contribution of *population density* was expected from the large differences in reaction strength between rural and urban areas (Figure 5A). The strong contribution of *unemployment rate* may be explained by the fact that the economy of high-unemployment districts is often concentrated in sectors that are more vulnerable to pandemic disruptions (retail, hospitality, and manual labor). During an outbreak, these jobs get shut down or drastically reduced, yielding a stronger reaction (23). Another explanation may be that high-unemployment districts often have a generally weak economy such that even without strict lockdowns, economic hardship enforces staying home. We note, however, that *unemployment rate* is moderately correlated with other predictors, particularly *income* and *voter turnout*, so its estimated effect may partly reflect the influence of these related variables. Furthermore, the regression coefficients of three other independent variables are also significantly different from zero: *agriculture, forestry, and fisheries* (*p* < 0.001, not multiple comparison corrected), *voter turnout* (*p* < 0.05, not multiple comparison corrected), and *income* (*p* < 0.05, not multiple comparison corrected). We tentatively propose explanations for the association between these three variables and the decrease in out-of-home duration. Our explanations are intended to motivate future investigation rather than establish causal relationships, and should be interpreted with caution given the exploratory nature of the analysis and the absence of multiple comparison correction. *Agriculture, forestry, fisheries* is an economic sector with little to no home office feasibility, where employees are generally expected to work on-site, thus limiting *reaction strength*. *Voter turnout* may be understood as a measure of political trust, and as such, high voter turnout enhances adherence to guidelines and consequently staying at home (24, 25). A higher average *income* has been associated with white-collar jobs, which allow for remote work more readily, increasing reaction strength (26). To conclude, the associations identified for these three variables are correlational, and direct causal interpretations are not warranted; they may rather be indicative of underlying social and economic conditions that shape mobility responses.

**Table 2:** Regression on reaction strength of either wave reveals the explanatory power of population density and job-related variables during the first, and the explanatory power of political variables during the second wave.

Variable	First wave standardized coefficient	Std. error	*p*-Value	Second wave standardized coefficient	Std. error	*p*-Value
Intercept	< 0.01	0.04	1	< 0.01	0.04	1
Population density	0.35	0.08	< 0.001^***^	0.17	0.07	0.009^**^
Unemployment rate	0.29	0.10	0.003^**^	0.40	0.11	< 0.001^***^
Income	0.23	0.10	0.02^*^	0.15	0.10	0.11
Voter turnout	0.16	0.07	0.03^*^	0.12	0.08	0.13
Manufacturing sector	0.11	0.06	0.05	−0.08	0.06	0.19
Small children in childcare	0.08	0.07	0.20	0.15	0.07	0.02^*^
Average age	0.02	0.07	0.81	−0.02	0.07	0.76
FDP	−0.04	0.06	0.47	−0.27	0.06	< 0.001^***^
SPD	−0.05	0.08	0.49	−0.19	0.08	0.02^*^
AfD	−0.09	0.08	0.27	−0.34	0.08	< 0.001^***^
Green party	−0.11	0.07	0.12	0.10	0.08	0.21
Agriculture, forestry, fisheries	−0.21	0.04	< 0.001^***^	−0.16	0.06	0.008^**^
Adj *R*.^2^	0.43			0.37		
AIC	928.32			967.92		
BIC	984.20			1,023.80		

Model outputs for regressand *reaction strength first* wave (middle columns) and *reaction strength second wave* (right columns). Asteriks indicate the significance levels with ^*^ representing *p* < 0.05, ^**^ representing *p* < 0.01, ^***^ representing *p* < 0.001.

**Table 3:** The variance inflation factors (VIF) of the all variables besides *unemployment rate* are all below the threshold of five, indicating only modest multicollinearity.

Variable	Variance inflation factor
Population density	3.13
Unemployment rate	5.80
Income	4.76
Voter turnout	3.17
Manufacturing sector	1.95
Small children in childcare	2.39
Average age	3.23
FDP	2.24
SPD	3.41
AfD	4.75
Green party	3.18
Agriculture, forestry, and fisheries	1.68

The VIF of *unmployment rate* indicates moderate multicollinearity, warranting further investigation.

Regressing on *reaction strength second wave* reveals a shift in explanatory power of independent variables, more concretely, away from labor and economic variables and toward political variables. The regression coefficients of three political party variables are now negative and significantly different from zero: *FDP, AfD* (both at a significance level of *p* < 0.001, not multiple comparison corrected), and SPD (significance level of *p* < 0.05, not multiple comparison corrected) (Figure 6C and Table 2 for detailed model output). A possible explanation may be that during the first wave, nonpharmaceutical interventions and communication strategies were implemented nationally, uniting society in an attempt to flatten the curve. These national implementations dictated home office feasibility and on-work site necessity clearly. During the second wave, this authority shifted more and more from the national to the federal state level, highlighting the different approaches of the political parties (and consequently their voters) to disease spread. The regression coefficient of *population density* is still statistically significant from zero (*p* < 0.01, not multiple comparison corrected), but comparable in magnitude to those of the political variables.

Together, our findings suggest that mobility responses shifted from being primarily driven by structural economic and demographic factors during the first wave to being increasingly shaped by election results and regional governance during the second wave.

### 2.5 Cross-district differences in peak incidence

Relevant for pandemic control, however, is not the strength of mobility reductions, but rather the peak height of the infection waves, which determines the maximal burden on the health care system. Peak height, in turn, depends on the decrease of mobility, but not exclusively. There certainly is a causal relationship, as a reduction of out-of-home duration limits contacts and consequently reduces infection opportunities and thus the effective reproduction number and peak height (10, 27). However, other factors also play a role, such as the local baseline reproduction number.

**Figure 7:**
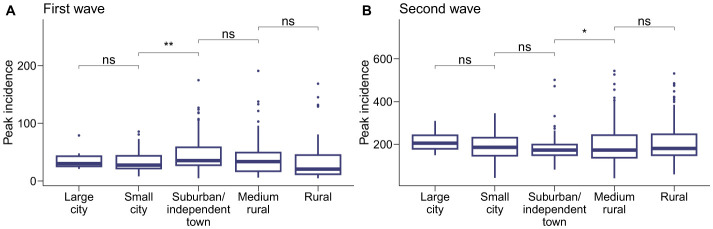
Stronger reaction strength does not necessarily yield smaller peak height. **(A)** For the first wave, the ANOVA test finds no significant difference in mean peak height (*p* = 0.124), while subsequent pair-wise *t*-test finds difference in peak height between small cities and suburban/independent towns significant. **(B)** For the second wave, peak incidence decreases slightly along the urban-rural gradient until it levels off for medium rural and rural districts (*p*-value ANOVA test = 0.05). In both panels, stars indicate that the *t*-test determined the means across district types to be statistically significant, with significance levels of ^**^ representing *p* < 0.01, ^*^ representing *p* < 0.05 and ns representing *p*>0.05. Peak incidence is defined as the 90th percentile of incidence during the corresponding wave.

A key indication is that during the first wave the peak height does not reveal an urban-rural gradient which we might have expected given the stronger decrease of mobility in urban districts (Figure 7A). Moreover, during the second wave, even though we observed the strongest reactions in cities, they also demonstrated the largest peak height (Figure 7B). The lack of urban-rural gradient suggests that rural areas may have a smaller baseline reproduction number. Differences in baseline reproduction may then, in turn, be traced back to demographic and socioeconomic factors.

To investigate these hypotheses, we used structural equation modeling, regressing on *peak incidence* of the first and the second wave separately (Figures 6C, D). As explanatory variables we considered the demographic, socioeconomic, and political variables of the previous section. *Reaction strength* entered the structural equation models as a mediator. If the peak incidence of either wave was driven solely by mobility reductions, only reaction strength should be significant, with other variables' effects mediated through it.

For both waves the reaction strength, i.e., our mobility measure, contributes to a decrease in peak incidence (Figures 6C, D, for the second wave, only at a significance level of *p* < 0.1. Note that the *x*-axis is flipped, for easier comparison with panels A and B). In contrast, population density has an increasing effect, counteracting the reaction strength, which might explain nearly the constant or increasing peak height from rural to urban districts. Indeed, urban areas must react more strongly in order to compensate for their higher population density, which often coincides with crowded public spaces, smaller living spaces, and a higher share of public transport, which all facilitate disease spread (28, 29). Otherwise, *agriculture, forestry, fisheries* is the only variable whose regression coefficient does not change its sign between the first and second wave. Thus, interpreting the influence of the other socioeconomic and political factors is difficult, despite them having a relatively high impact during both waves. Similarly as for the regression on reaction strength, this might be a indication that the context of the first and second wave was different, from mitigation being driven more by Germany-wide interventions and restrictions during the first wave, to more federal state wide interventions and voluntary cautious behavior during the second wave. Taken together, these results show that our measure of mobility is only one of the factors determining the COVID-19 spread and spreading models and predictions may be improved by taking into account the composite of mobility and other environmental factors.

## 3 Discussion

In this work, we quantified the change in local mobility in Germany's 400 districts during the first year of the COVID-19 pandemic year using out-of-home durations derived from anonymized mobile phone data. For each week, our hierarchical Bayesian model decomposes the out-of-home duration into a product of a baseline out-of-home duration, a disease factor based on the local and national COVID-19 incidence, and three disease-independent factors (temperature, school vacations, and public holidays). Three findings stand out: first, the model successfully reproduces both the timing and the magnitude of the major reductions in out-of-home duration across districts. This required incorporating a disease factor with diminishing impact over time, reflecting pandemic fatigue. Second, national incidence carries more weight in explaining mobility behavior than local incidence. Third, districts' reductions in out-of-home duration in response to disease spread varied significantly across space, decreasing systematically from large cities to rural areas.

Our Bayesian inference model attributes most of the reduction in out-of-home duration to disease spread [median reduction first wave: −23%, IQR: (−27%, −20%), median reduction second wave: −18%, IQR: (−21%, −15%)], secondary effects to temperature (seasonal differences of 11%) and only modest reduction to calendar effects [school vacations [−4%], public holidays [−3%]), aligning with evidence that policy and perceived risk jointly drive mobility changes (10, 27, 30). Our framework, however, cannot fully disentangle the drivers behind the impact of disease spread on behavior. The disease factor, which captures the reaction to incidence, combines government-mandated and voluntary responses that co-vary with incidence and media coverage (31, 32), which must be considered when interpreting our results. Importantly, to infer the out-of-home duration successfully, the disease factor has to incorporate that the reduction in out-of-home duration resulting from disease spread weakens over time. This gradual relaxation is consistent with survey-based evidence on declining adherence (33, 34), though the underlying mechanisms—habituation, economic pressure, poor communication—need further investigation and may vary between subpopulations.

The stronger influence of national compared to local incidence suggests that information and policy signals were integrated on a national level. This is plausible in Germany, where nationwide announcements and broadly coordinated measures dominated in 2020 (35). The heavy weight of national incidence also reflects that mobility behaviors are coupled across districts through commuting and media consumption. Nevertheless, local incidence does play a minor role, with its influence increasing from urban to rural districts: Large cities dominate the national incidence rate, so their local rates align closely with it, while rural districts show larger deviations from the national average. These discrepancies may motivate rural residents to react more strongly to disease spread in their communities. However, we do not claim this weight to be universal. In settings with highly decentralized policies or localized outbreaks, local incidence may matter more. Understanding how people weight varying signals remains an open problem with implications for the timing and targeting of NPIs and communication strategies.

We found districts to vary significantly in how much they reduced out-of-home duration in response to disease spread (for ANOVA test *p* < 0.0001, for subsequent pairwise *t*-tests *p* < 0.001), with reductions decreasing systematically from large cities to rural areas. The observed urban-rural gradient in out-of-home duration reduction can be motivated by higher vulnerability and baseline contact opportunities in cities (14, 15), yet density alone cannot fully account for the variation. Regional clusters indicate that inter-district connectivity, sectoral composition, and early outbreak histories also matter (3). Political and social context likely contributes as well: our governmental trust-proxy (voter turnout) correlates with stronger reductions, echoing work linking trust in government and institutions to adherence (8, 33). Differences in home office feasibility by occupation further support larger potential reductions in higher-income, service-oriented urban districts (26). These observations are supported by our regression, where population density and unemployment rate were the dominant correlates for both waves, followed by modest contributions of additional socio-economic and political covariates (first wave: voter turnout, industries with little home office feasibility, second wave: voting shares).

There are three caveats one has to keep in mind when interpreting our results. First, smartphone-based measures can under-represent specific demographics (e.g., the very old or young) and essential workers, potentially biasing urban-rural comparisons (36). Second, district-level covariates risk ecological fallacy. As coefficients are estimated on between-district variation, their magnitudes also reflect how much each covariate varies across districts. Variables with limited between-district variation (even if heterogeneous within districts) will yield smaller standardized regression coefficients. Individual-level data could improve model accuracy and help understand equity implications. Third, the inferred importance of population density is robust and aligns with known structural differences, but the socio-economic factors with smaller coefficients—income, unemployment, childcare, voter turnout—may be proxies for latent constructs (job mix, employer practices, information environments). Natural experiments that shift these factors, for instance change in home office feasibility or closure of childcare facilities, would be needed to isolate mechanisms (31, 32). As a result, our regression and structural equation model should not be interpreted as causal.

A central methodological choice we made is the mobility measure. We deem the out-of-home duration an appropriate choice, as it directly tracks time potentially spent in shared physical spaces and is comparable across districts and weeks. This contrasts with distance-based measures, trip counts, or venue visitation indices, which may strongly correlate with out-of-home duration, but may also capture different behavioral facets (37–39). Out-of-home duration aggregates across trip purposes without requiring venue classification and maps naturally to contact time in many settings, though it does not distinguish crowded from sparse environments, indoor from outdoor time, or clustering at specific locations—all of which influence transmission dynamics (36, 40, 41). This limitation becomes evident in the imperfect link between reductions in out-of-home duration and peak incidence: Within-own household transmissions, crowding in essential workplaces, and lack of other self-protective behaviors (e.g., mask-wearing) can sustain spread even when time away from home declines (42). Additionally, cities may need larger reductions to offset inherently higher contact opportunities linked to crowding in places ranging from home to public transport vehicles (41). This underscores the need to integrate a mobility quantity with context (who, where, and how crowded) to improve predictive value. Combining duration with occupancy and indoor-air proxies, and modeling network structure explicitly are promising directions (3, 40).

Traditionally, smartphone-derived mobility data tend to oversample younger, urban, and higher-income populations, which raises questions about the representativeness of our mobility measure. In Germany, however, this concern is mitigated by high smartphone penetration rates distributed broadly across age groups, income levels, and urban-rural divides [smartphone penetration rate of 97% in 2020, (43)]. Moreover, our previous work has shown that out-of-home duration remains a strong predictor of COVID-19 incidence growth despite these potential demographic limitations, suggesting that the mobility signal is behaviorally meaningful even when its source population is not perfectly representative (4, 16).

Some aspects of our modeling approach present opportunities for improvement. For instance, our disease factor combines local and national incidence for Germany but does not include neighboring districts' or cross-border epidemiological dynamics, which may affect border regions (44). Furthermore, we observe that minimal out-of-home duration in early March 2020 preceded the incidence peak; incorporating additional drivers, such as media, risk-perception signals or international incidence, may improve timing, especially at the start of waves (33, 34, 37, 45).

Overall, our findings offer valuable practical implications for pandemic preparedness. The near real-time availability of out-of-home duration makes it a crucial asset for outbreak response. Our results validate its use as a behavioral proxy for population response, complementing slower epidemiological indicators (37). The observed urban-rural gradient suggests opportunities for locally tailored interventions and communication strategies: dense cities may require earlier or stronger measures to achieve comparable risk reduction, while rural areas may benefit from strategies that address different job mixes and transport patterns (14, 15).

## 4 Methods

### 4.1 Input Variables

#### 4.1.1 COVID-19 case numbers

**Figure 8:**
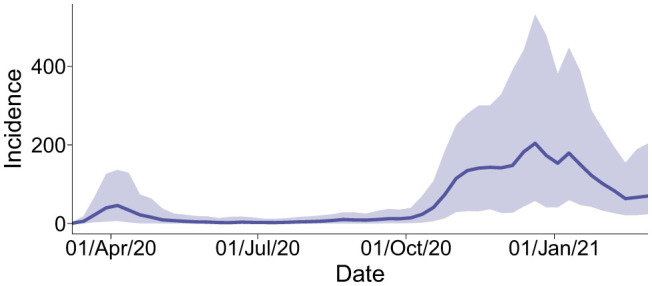
7-day incidence per 100,000 inhabitants: the first COVID-19 wave was largely suppressed in Germany, higher incidences are reached during the second wave. Two local maxima occur: due to the first wave (spring 2020) and the second wave (winter 2020/2021). Peak heights differ widely across districts, with some districts experiencing almost no first wave. Line represents the mean across districts, ribbons correspond to 95% interpercentile range.

**Figure 9:**
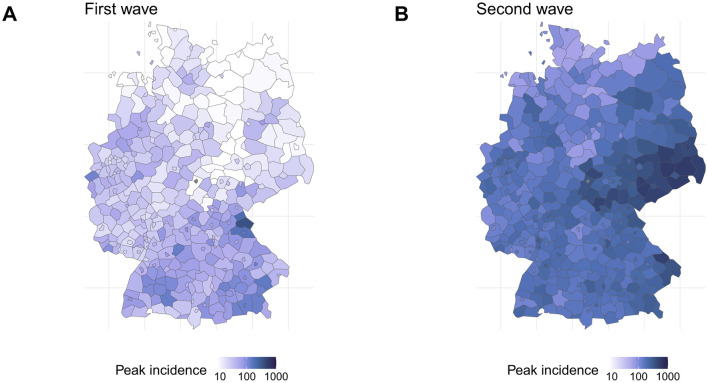
Spatial depiction of COVID-19 incidences. **(A)** Southern/western Germany dominated the first wave. **(B)** Eastern Germany (near Czechia) had the highest incidence rates in the second wave. For each district, the 90th percentile of the first and second wave are depicted.

In Germany, the first COVID-19 case was detected on January 27th, 2020. Case numbers began to steeply rise in March 2020, reaching the first local maximum between March 29th, 2020 and April 12th, 2020, depending on the district (Figure 8). Across districts, the average of the local maximum 7-day incidence/100,000 was 54 [median of maxima: 44, IQR: (27,67)]. The height of the initial wave differed greatly across districts, with some districts experiencing no first wave at all, and the highest outbreaks being observed in southern Germany and parts of North Rhine-Westphalia (Figure 9A). From the maximum onward, case numbers decreased and remained low throughout summer. Cases began to rise again beginning mid-September, reaching a second peak around the turn of the year 2020/2021. Again, second wave heights differed greatly across districts, with a mean maximum of 251 [median: 223, IQR: (181, 294)]. Eastern Germany, specifically the districts close to Czechia, endured the highest second waves (Figure 9B). Overall, during the first pandemic year, Germany experienced two pandemic waves, one in the spring and one in the fall and winter, with large differences between districts.

#### 4.1.2 Temperature

**Figure 10:**
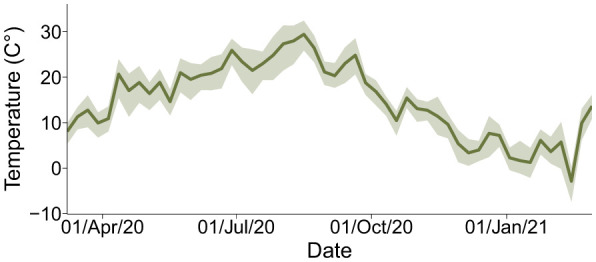
Temperature follows a seasonal pattern, with highs in summer and lows in winter. For each district, the weekly average of the daily maximum temperature is used. The global maximum temperature occurs mid-August 2020. From then onward, temperature steadily decreases until it reaches a global minimum in February 2021. Line represents the mean across districts, ribbons correspond to 95% interpercentile range.

In Germany, weather influences the out-of-home duration throughout the year, with warmer temperatures motivating individuals to spend extended periods outside their homes, whereas lower temperatures promote staying at home (18, 19). Temperatures hereby follow a seasonal pattern: During the winter months, November to February, temperatures are low, partly dropping below the freezing point, while the summer months, June to August, bring high temperatures up to 30°C (Figure 10). During the study period, the maximal average temperature was recorded mid-August 2020, whereas the minimal average temperature was recorded in February 2021. For our analysis, we consider the weekly average of the daily maximum temperature. Whenever temperature data is unavailable for a district, we consider the average temperature of all bordering districts for which temperature data is available. In sum, weather affects the out-of-home duration, with higher temperatures encouraging time spent outside one's home and lower temperatures promoting staying indoors, following Germany's seasonal pattern of winter lows (below freezing point) and summer highs (up to 30°C).

#### 4.1.3 School vacation

**Figure 11:**
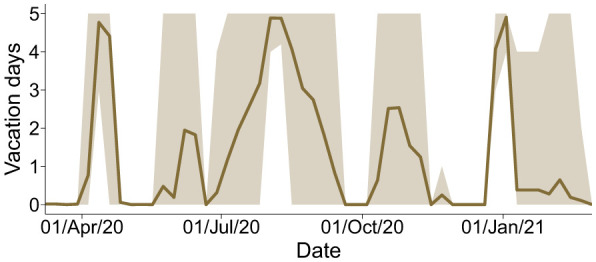
Summer break is the most extensive vacation period in Germany, shorter periods in the spring and winter. For each week, the number of school vacation days (from Monday until Friday) is summed up. Vacations differ between federal states; plotted here (as a line) is the mean across federal states. Most federal states have four breaks: spring, summer, fall, and winter, with summer vacation (July–September) representing the longest break period. Ribbons correspond to 95% interpercentile range.

School vacations decrease the out-of-home duration through two mechanisms: directly by keeping children from going to school and indirectly by requiring parents to take vacation days or to work remotely to look after their children. In Germany, school vacations differ by federal state, with most states having spring, summer, fall, and winter breaks. For each week, we sum up the number of school vacation days (Figure 11). As children in Germany go to school from Monday until Friday, the maximum number of school vacation days per week is five. Summer vacation (between July and September), with a duration of 6 weeks in most federal states, represents the longest break period. Overall, school vacations decrease the weekly out-of-home duration by keeping children home from school and prompting parents to take vacation days, with timing varying across German federal states due to different vacation schedules.

#### 4.1.4 Public holidays

**Figure 12:**
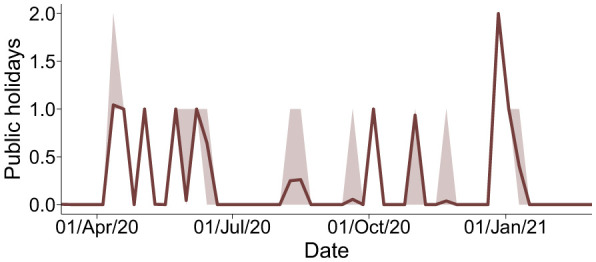
With the exception of Christmas week, only one public holiday occurs per week. For each week, the number of public holidays that fall on a business day are summed up. The majority of public holidays occurs nation-wide. Only Christmas week has two public holidays; all other weeks contain a single holiday or none. Line represents the mean across federal states, ribbons correspond to 95% interpercentile range.

Public holidays that fall on business days decrease the out-of-home duration both directly by exempting many employees from work and indirectly by encouraging employees to take additional vacation days on the business day preceding and/or following the public holiday. This decreases the out-of-home duration on the public holiday itself and the days adjacent to the public holiday, which, in turn, decreases the weekly average out-of-home duration. In Germany, some public holidays are national holidays (including Easter Monday, New Year's Eve, and New Year's Day), while others differ by federal state (including International Women's Day and All Saints' Day). For each week, we sum up the number of public holidays that fall on a business day (Figure 12). During the study period, Christmas week was the only week with two public holidays. Overall, public holidays decrease the weekly out-of-home duration both directly through work-free days and indirectly through adjacent vacation days, with effects varying by region due to differences in federal holiday schedules.

### 4.2 Output variable: Out-of-home duration

**Figure 13:**
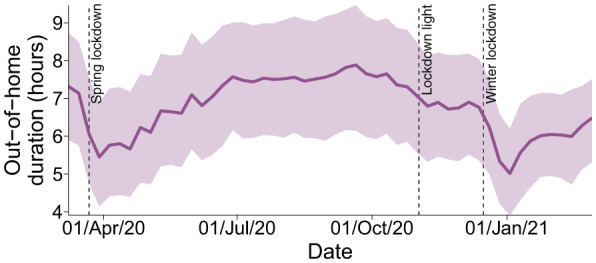
In all districts, the out-of-home duration decreases sharply in spring and winter 2020/2021. Sharp decreases can be observed in March and December 2020. Out-of-home duration does not plateau at the local minima, but immediately rises again. During the summer 2020, out-of-home durations exceeding pre-pandemic levels from early March are observed. The dashed lines indicate the introduction of the different German lockdowns. The first far-reaching contact restrictions were introduced on March 22nd, 2020. The second set of national restrictions, the so-called *lockdown light*, were introduced on November 2nd, 2020, yielding a partial lockdown. The third lockdown was implemented on December 16th, 2020 and included shop, hairdresser, and school closures. Line represents the mean across districts, ribbons correspond to the 95% interpercentile range.

The out-of-home duration is a measure of local mobility, describing the amount of time (in hours and per day) an average person spends outside their home. This duration fluctuated considerably throughout the study period, with the first change being observed in the second week of March 2020, when the out-of-home duration sharply decreased (Figure 13). Contact reductions were introduced in the week ending March 23rd, 2020, the week the out-of-home duration reached a local minimum. Rather than plateauing at this low point, the out-of-home duration began to increase immediately, notably before the first governmental relaxations (weeks ending May 4th, 2020 and May 11th, 2020). Throughout the summer of 2020, the out-of-home duration exceeded pre-pandemic levels from early March 2020, until mid-September, when another decrease in out-of-home duration is observed. The so-called *lockdown light*, introduced on November 2nd, 2020, stopped the decreasing trend and lead to a plateau (potentially due to hasty Christmas-shopping and the looming stronger lockdown). This plateau was followed by a dramatic decrease coinciding with more restrictive contact measures introduced on December 16th, 2020. During the winter of 2020/2021, the out-of-home duration fell to levels comparable to those observed during the initial COVID-19 wave in March 2020. Altogether, the out-of-home fluctuated throughout the study period, declining sharply during lockdowns in March 2020 and winter 2020/2021, but exceeding pre-pandemic levels during summer 2020, with changes often occurring before rather than after policy implementations.

We chose the somewhat unconventional out-of-home duration as our mobility measure based on two complimentary lines of reasoning. First, in our large-scale agent-based modeling framework developed during the COVID-19 pandemic, out-of-home duration served as a central input variable and consistently proved influential in shaping simulated infection dynamics (6). This strong effect was further supported by subsequent work, in which we demonstrated a quadratic relationship between reductions in out-of-home duration and infection growth (4). Given this strong directional effect, this work deliberately reverses the relationship, treating out-of-home duration as the outcome to be explained. Second, we have previously examined alternative mobility metrics (including traveled distance and the proportion of mobile persons), finding that these strongly correlate with out-of-home duration, suggesting no analytical advantage in substituting them (see Supplementary section 5.1).

### 4.3 Bayesian hierarchical model

**Figure 14:**
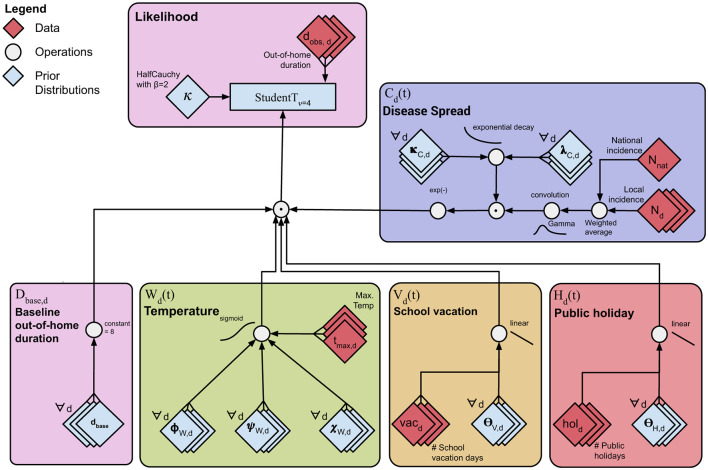
Statistical model overview.

The model aims to infer the out-of-home duration *D*_*d*_(*t*) for each German district *d*∈{1, …, 400} for each week *t*∈{1, …, 52}, spanning the period from March 2020 until March 2021. We assume that the out-of-home duration *D*_*d*_(*t*) can be modeled as the product of a baseline out-of-home duration *D*_base, *d*_, multiplied by a disease factor dependent on local and national COVID-19 spread *C*_*d*_(*t*) and three disease-independent factors (temperature *W*_*d*_(*t*), school vacation *V*_*d*_(*t*), and public holidays *H*_*d*_; Figure 14 for graphical overview and Table 4 for summary of prior distributions). We assume that these factors are multiplicative (i.e., additive in log-space) as changes in one factor do not yield changes in another and as we assume that their effects can be expressed as multiples of a base level. The multiplicative inference model reads:


$$\begin{array}{l} D_{d}\left(t\right)=D_{\text{base},d}\cdot W_{d}\left(t\right)\cdot V_{d}\left(t\right)\cdot H_{d}\left(t\right)\cdot C_{d}\left(t\right) \end{array}$$

The different factors are discussed in the subsequent subsections.

**Table 4:** Prior distributions and their meaning in our Bayesian inference model.

Parameter	Prior distribution	Rationale/Meaning
Baseline out-of-home duration *D*base, *d*		
$\mu_{\text{base}}^{\theta }$	N(0,0.2)	Used to determine how much *D*base, *d* in district *d* deviates from the assumed 8 h of baseline out-of-home duration
$\sigma_{\text{base}}^{\theta }$	HalfNormal(0.3)	Used to determine variance across districts in deviating from the assumed 8 h of baseline out-of-home duration, weakly-informative
Temperature factor *Wd*		
$\mu_{W}^{\phi }$	HalfCauchy(1)	Determines if temperature impact out-of-home duration. For values close to 0, the impact of temperature vanishes
$\sigma_{W}^{\phi }$	Exp(10)	Determines variance across districts in impacting out-of-home duration, weakly-informative
$\mu_{W}^{\psi }$	N(15,3)	Determines shift of sigmoid function. Based on previous work, in which we explored the threshold temperature above which individuals move their activities outside (4, 6)
$\sigma_{W}^{\psi }$	Exp(10)	Determines variance in shift across districts, weakly-informative
$\mu_{W}^{\chi }$	N(ln(4),0.5)	Determines slope of sigmoid function, weakly-informative
$\sigma_{W}^{\chi }$	Exp(10)	Determines variance in slope across districts, weakly-informative
School vacation factor *Vd*		
$\mu_{V}^{\theta }$	N[0.8,1]	Amplitude of impact of vacation factor, non-informative
$\sigma_{V}^{\theta }$	Exp(10)	Determines variance in amplitude across districts, weakly-informative
Public holiday factor *Hd*		
$\mu_{H}^{\theta }$	U[0.9,1]	Amplitude of impact of public holiday factor, non-informative
$\sigma_{H}^{\theta }$	HalfNormal(0.25)	Determines variance in amplitude across districts
Disease factor *Cd*		
$\mu_{C}^{\phi }$	N(ln(1.5),0.25)	Determines initial quantity of exponential decay, weakly-informative
$\sigma_{C}^{\phi }$	Exp(10)	Determines variance in initial quantity across districts, weakly-informative
$\mu_{C}^{\psi }$	N(30,1)	Determines exponential decay constant, weakly-informative
$\sigma_{C}^{\psi }$	HalfNormal(0.1)	Determines variance in exponential decay constant across districts
$z_{C,d}^{\omega }$	$N\left(\mu_{C}^{\omega },1\right)$	Determination of weight of normalized local incidence
$\mu_{C}^{\omega }$	N(0,0.5)	Determines mean of weight of normalized local incidence
$\mu_{C}^{G}$	N(3,1)	Determines mean of gamma memory distribution
$\sigma_{C}^{G}$	HalfNormal(0.25)	Determines variance in mean across districts
$\alpha_{C}^{G}$	LogNormal(ln(3),0.125)	Used (in combination with μ_*C, d*_) to determine standard deviation of gamma memory distribution. Based on the assumption that districts differ little in their memory.

#### 4.3.1 Baseline out-of-home duration

The baseline out-of-home duration *D*_base, *d*_ differs across districts: Districts with high concentration of tradespeople, healthcare professionals, hospitality workers, or with a large share of employees working in manufacturing or mining, typically have longer out-of-home durations as employees need to by physically present onsite, forcing them to commute to work and leave their home. In contrast, districts dominated by remote work, with high shares of retirees or high unemployment rates, may show lower baseline out-of-home duration. Finally, commuting time is typically longer in rural than in urban areas, as employees in rural areas need to cross greater distances to reach their worksite, increasing out-of-home duration (46). The baseline out-of-home duration *D*_base, *d*_ encapsulates this district-dependency, representing the out-of-home duration during a week with no disease spread, no public holidays or school vacation, and average temperatures:


$$\begin{array}{l} \;D_{\text{base},d}=\theta_{\text{base},d}\cdot 8,\\ \text{where }\theta_{\text{base},d}=\text{exp}\left(\mu_{\text{base}}^{\theta }+z_{\text{base},d}\cdot \sigma_{\text{base}}^{\theta }\right),\\ \;z_{\text{base},d}\sim N\left(0,1\right),\\ \;\mu_{\text{base}}^{\theta }\sim N\left(0,0.2\right),\\ \;\sigma_{\text{base}}^{\theta }\sim \text{HalfNormal}\left(0.3\right). \end{array}$$

The underlying assumption of *D*_base, *d*_ is that the baseline out-of-home duration fluctuates around 8 h, modeled hierarchically across districts, reflecting the typical daily work time of a full-time employee in Germany.

#### 4.3.2 Temperature

Weather, here represented by the weekly average of daily maximum temperature , increases out-of-home duration during the warmer months (typically April through September) and decreases out-of-home duration during the colder months (typically October through March). We model this effect using a sigmoid function, which captures the saturation of weather's effect at the temperature extremes. For Germany specifically, a sigmoid function provided the best fit for the temperature effect, consistent with the country's climatic and cultural context. German summers rarely reach temperatures extreme enough to drive people indoors, and air conditioning remains uncommon in German households; unlike in hotter climates, where cooling infrastructure can suppress mobility at high temperatures. Alternative functional forms for the weather effect are discussed in Supplementary section 5.3. The inflection point of the sigmoid is modeled such that it takes on a value of 1, so that a week with average annual temperature neither in- nor decreases out-of-home duration. All free variables, the effect size ϕ_*W, d*_, the temperature offset ψ_*W, d*_, and the steepness χ_*W, d*_ are modeled hierarchically across districts.


$$\begin{array}{l} W_{d}\left(t\right)=\phi_{W,d}\cdot \left(\frac{1}{1+exp\left(-\left(t_{\text{max},d}(\text{t})-\psi_{W,d}\right)/\chi_{W,d}\right)}\right)\\ \;+(1-\phi_{W,d}/2),\\ \phi_{W,d}=\mu_{W}^{\phi }+\sigma_{W}^{\phi }\cdot z_{W,d}^{\phi },\\ z_{W,d}^{\phi }\sim N\left(0,1\right),\\ \mu_{W}^{\phi }\sim \text{HalfCauchy}\left(1\right),\\ \sigma_{W}^{\phi }\sim \text{Exp}\left(10\right),\\ \psi_{W,d}=\mu_{W}^{\psi }+\sigma_{W}^{\psi }\cdot z_{W,d}^{\psi },\\ z_{W,d}^{\psi }\sim N\left(0,1\right),\\ \mu_{W}^{\psi }\sim N\left(15,3\right),\\ \sigma_{W}^{\psi }\sim \text{Exp}\left(10\right),\\ \chi_{W,d}=\text{exp}\left(\mu_{W}^{\chi }+\sigma_{W}^{\chi }\cdot z_{W,d}^{\chi }\right),\\ z_{W,d}^{\chi }\sim N\left(0,1\right),\\ \mu_{W}^{\chi }\sim N\left(\text{ln}\left(4\right),0.5\right),\\ \sigma_{W}^{\chi }\sim \text{Exp}\left(10\right). \end{array}$$

#### 4.3.3 School vacation

School vacations typically decrease out-of-home duration because students no longer attend schools and parents often take vacation days or work remotely to care for their children. The more business days are school vacation days, the stronger the reduction in week *t*.


$$\begin{array}{l} \;V_{d}\left(t\right)=\frac{\theta_{V,d}-1}{7}\cdot {\text{vac}}_{d}\left(t\right)+1,\\ \text{where }\theta_{V,d}=\mu_{V}^{\theta }+\sigma_{V}^{\theta }\cdot z_{V,d},\\ \;z_{V,d}\sim N\left(0,1\right),\\ \;\mu_{V}^{\theta }\sim U\left[0.8,1.0\right],\\ \;\sigma_{V}^{\theta }\sim \text{Exp}\left(10\right). \end{array}$$

Here, vac_*d*_(*t*) is the number of school vacation days during week *t*. In Germany, students go to school from Monday to Friday. Consequently, vac_*d*_(*t*) may take up integer values between 0 and 5. If every school day of week *t* is a vacation day, then $V_{d}\left(t\right)=\frac{5}{7}\theta_{V,d}+\frac{2}{7}$, reflecting that only the out-of-home duration on the five school days is affected by the school vacation, while the out-of-home duration on the weekend remains unaffected.

#### 4.3.4 Public holidays

Public holidays that fall on business days decrease out-of-home duration both by exempting employees from work and by incentivizing them to extend their time off by taking additional vacation days before and/or after the holiday.


$$\begin{array}{ll} H_{d}\left(t\right) & =\frac{\theta_{H,d}-1}{7}\cdot {\text{hol}}_{d}\left(t\right)+1, \end{array}$$

where


$$\begin{array}{l} \theta_{H,d}=\mu_{H}^{\theta }+\sigma_{H}^{\theta }\cdot z_{H,d},\\ z_{H,d}\sim N\left(0,1\right),\\ \mu_{H}^{\theta }\sim U\left[0.9,1\right],\\ \sigma_{H}^{\theta }\sim \text{HalfNormal}\left(0.25\right). \end{array}$$

Here, hol_*d*_(*t*) is the number of public holidays during week *t*. In Germany, the majority of full time employees works from Monday to Friday. Consequently, hol_*d*_(*t*) may take on integer values between 0 and 5. If every business day of week *t* is a public holiday, then $H_{d}\left(t\right)=\frac{5}{7}\theta_{H,d}+\frac{2}{7}$, reflecting that only the out-of-home duration on the business days is affected by the publich holiday, while the out-of-home duration on the weekend remains unaffected.

#### 4.3.5 Disease spread

We assume that disease spread has a decreasing effect on out-of-home duration: High case numbers motivate more stringent NPIs and increased risk perception which both, in turn, lead to smaller out-of-home activity and consequently decrease out-of-home duration. The disease factor *C*_*d*_(*t*) takes the national and local COVID-19 7-Day incidence rate as input and is computed in four steps:

1. First, assuming that both local and national disease spread influence local mobility behavior, a weighted average of the normalized local COVID-19 incidence rate *N*_*d*_(*t*) and normalized national COVID-19 *N*_nat_(*t*) incidence rate is computed.
2. Second, assuming that out-of-home duration in week *t* is not only instantaneously influenced by disease spread in week *t*, but also by disease spread in the recent past, the weighted average is convolved with a Gamma distribution.
3. Third, assuming that the population's perception of risk decreases over time as pandemic fatigue sets in, the convolved case numbers are multiplied with an exponential decay. This ensures that the same level of case case numbers reduces the out-of-home duration less at a later point in time.
4. Fourth, as we assume it has a multiplicative effect, we take the negative exponential of the convoluted and exponentially decayed case numbers. This also ensures a smaller than 1 multiplicative effect for positive case numbers.

Together the disease factor reads:


$$\begin{array}{l} C_{d}\left(t\right)=exp(-z_{C,d}\left(t\right)\cdot \int_{-\infty }^{t}(\omega_{C,d}\cdot N_{d}\left(t\right)+\left(1-\omega_{C,d}\right)\\ \cdot N_{\text{nat}}\left(t)\right)\cdot G(t-t^{\prime },\mu_{C,d},\sigma_{C,d})dt^{\prime }), \end{array}$$

where κ_*C, d*_ is the exponential decay:


$$\begin{array}{l} z_{C,d}\left(t\right)=\kappa_{C,d}\cdot exp\left(-t/\lambda_{C,d}\right),\\ \;\kappa_{C,d}=\text{exp}\left(\mu_{C}^{\phi }+\sigma_{C}^{\phi }\cdot z_{C,d}^{\kappa }\right),\\ \;z_{C,d}^{\kappa }\sim N\left(0,1\right),\\ \;\mu_{C}^{\phi }\sim N\left(\text{ln}\left(1.5\right),0.25\right),\\ \;\sigma_{C}^{\phi }\sim \text{Exp}\left(10\right),\\ \;\lambda_{C,d}=\text{softplus}\left(z_{C,d}^{\lambda }\right),\\ \;z_{C,d}^{\lambda }\sim N\left(\mu_{C}^{\psi },\sigma_{C}^{\psi }\right),\\ \;\mu_{C}^{\psi }\sim N\left(30,1\right),\\ \;\sigma_{C}^{\psi }\sim \text{HalfNormal}\left(0.1\right). \end{array}$$

ω_*C, d*_ is the local vs. national weighting:


$$\begin{array}{ll} \omega_{C,d} & =1/\left(1+exp\left(-z_{C,d}^{\omega }\right)\right),\\ z_{C,d}^{\omega } & \sim N\left(\mu_{C}^{\omega },1\right),\\ \mu_{C}^{\omega } & \sim N\left(0,0.5\right). \end{array}$$

And μ_*C, d*_ and σ_*C, d*_ the mean and standard deviation of the Gamma kernel:


$$\begin{array}{ll} \mu_{C,d} & =\text{softplus}\left(\mu_{C}^{G}+\sigma_{C}^{G}\cdot z_{C,d}^{\text{Γ}}\right),\\ z_{C,d}^{\text{Γ}} & \sim N\left(0,1\right), \end{array}$$

$$\begin{array}{l} \mu_{C}^{G}\sim N\left(3,1\right),\\ \sigma_{C}^{G}\sim \text{HalfNormal}\left(0.25\right),\\ \sigma_{C,d}=\mu_{C,d}/\sqrt{\alpha_{C}^{G}},\\ \alpha_{C}^{G}\sim \text{LogNormal}\left(\text{ln}\left(3\right),0.125\right). \end{array}$$

#### 4.3.6 Likelihood

Finally, we define a goodness of fit of our model to the observed out-of-home duration. The likelihood is hereby modeled using a Student's *t*-distribution as this allows for some outliers due to its heavier tails. The likelihood reads


$$\begin{array}{ll} L_{d}\left(t\right) & \sim {\text{StudentT}}_{\nu =4}\left(D_{d}\left(t\right),\sigma_{L}\right),\\ \;\sigma_{L} & \sim \text{HalfCauchy}\left(2\right). \end{array}$$

### 4.4 Inference

We use the package PyMC (47) to build the model and the NUTS sampler for inference (48) via the library Nutpie (49). We use 2,000 tuning steps and 1,000 draws in four parallel chains. We made sure the chains converged, as the R-hat value is below 1.07 for all variables.

### 4.5 Linear model explaining cross-district variation in reaction strength

First analysis reveals significant differences in reaction strength between district types (Section 2.4). However, district type alone does not suffice to fully explain the observed differences. To explore the differences in reaction strength further, we conduct a multi-step analysis separated by wave. To this end, we define *reaction strength first wave* as the integral over the exponential decay function for the first 13 weeks of the study period, ranging from the week ending March 8th, 2020 until the week ending May 31st, 2020, while we define *reaction strength second wave* as the integral over the exponential decay function for the final 26 weeks of the study period, ranging from the week ending September 6th, 2020 until the week ending February 28th, 2021. For each wave we follow the same four step approach:

1. Exploratory analysis of correlation coefficients of *reaction strength* and *population density* and nineteen additional demographic, socioeconomic, and political explanatory variables (Table 5 for considered variables).
2. Exhaustive search for each number of variables between 1 and 20. For each number of variables, we chose the subset with the highest adjusted *R*^2^.
3. Model selection across subset sizes using (adjusted and predicted) *R*^2^, Mallow's *C*_*p*_, AIC, and BIC.
4. Regression on *reaction strength* to determine the demographic, socioeconomic, and political variables' ability to explain observed variance in *reaction strength*.

**Table 5:** Demographic, socioeconomic, and political variables considered in the regression and structural equation models differ across districts.

Variable	Description	Mean	Median	IQR
Population density	Extrapolation of inhabitants per km^2^ on December 31st, 2022 based on 2011 census	544.1	204.5	(118.0, 686.0)
Income	Primary income of private households, including private non-profit organizations in 2020, in EURO	27,811.9	27,842.5	(24,679.0, 31,021.3)
Average age	Average age of the population on December 31st, 2020	45.2	45.0	(44.0, 46.4)
65+ year olds	Share of people aged 65 and older in the total population in 2020, in %	22.3	22.0	(20.5, 23.9)
Small children in childcare	Share of children under the age of three in daycare facilities/daycare centers in this age group in 2020, in %	33.1	29.9	(24.5, 36.3)
Unemployment rate	Unemployment rate relative to the total civilian labor force (sum of working population and registered unemployed persons) in 2020, annual average, in %	5.5	5.2	(3.8, 6.6)
Employment rate	Division of the number of employees subject to social insurance contributions on June 30th, 2020 by the population aged 15-64 on December 31 of the previous year, in %. The 2020 *employment rate* was unavailable for the following districts: Suhl, Wartburgkreis, Schmalkalden-Meiningen, Ilm-Kreis, Sonneberg, Saalfeld-Rudolstadt. For these districts, we use the 2021 *employment rate*	62.2	62.7	(60.0, 65.3)
Service sectors	Proportion of employed persons in service sectors in 2020, in %	70.7	70.4	(63.7, 77.7)
Manufacturing sector	Proportion of employed persons in the manufacturing sector in 2020, in %	27.4	26.4	(20.3, 34.1)
TTHIC sectors	Proportion of employed persons in the trade, transport, hospitality, information, and communication sector in 2020, in %	24.4	24.0	(21.8, 26.9)
Finance sector	Proportion of employed persons in financial, insurance, and business services, real estate and housing sector in 2020, in %	14.1	13.1	(11.2, 15.7)
Construction	Proportion of employed persons working in construction in 2020, in %	6.7	6.6	(5.0, 8.3)
Agriculture, forestry, fisheries	Proportion of employed persons in agriculture, forestry and fisheries in 2020, in %	1.9	1.6	(0.5, 2.9)
Voter turnout	Federal election turnout in 2017 in districts, in %	75.9	76.1	(73.6, 78.4)
CDU	Share of votes for political party *Christian Democratic Union of Germany* (CDU) in 2017 federal election, in %	34.3	33.9	(30.0, 38.7)
SPD	Share of votes for political party *Social Democratic Party of Germany* (SPD) in 2017 federal election, in %	20.1	19.2	(15.0, 25.0)
AfD	Share of votes for political *Alternative for Germany* (AfD) in 2017 federal election, in %	13.4	12.0	(9.8, 15.2)
FDP	Share of votes for political party *Free Democratic Party* (FDP) in 2017 federal election, in %	10.1	9.9	(8.3, 11.5)
Left party	Share of votes for political party *The Left* in 2017 federal election, in %	8.8	6.8	(5.7, 10.2)
Green party	Share of votes for political party *Alliance 90/The Greens* (green party) in 2021 federal election, in %	8.1	7.3	(5.4, 10.5)

Due to the differences in scale, all variables are standardized for the regressions.

#### 4.5.1 Exploratory analysis

**Figure 15:**
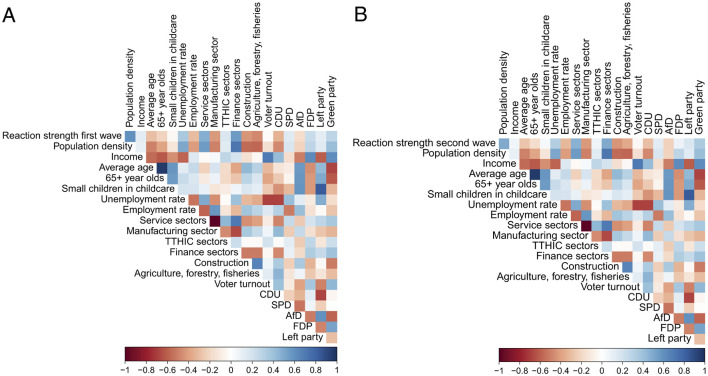
*Reaction strength* of either wave is strongly correlated with *population density* and employment sectors which easily or not at all permit remote work. *Reaction strength* correlates most strongly positively with *population density, service sectors*, and *finance sector*. *Agriculture, forestry, fisheries, manufacturing sector*, and *construction* are negatively correlated with *reaction strength*. **(A)** Correlation coefficients of *reaction strength first wave* and the 20 chosen explanatory variables. **(B)** Correlation coefficients of *reaction strength second wave* and the 20 chosen explanatory variables. Only the first row differs from **(A)**.

In the first analysis step, we consider linear correlations between *reaction strength first wave* and *reaction strength second wave* on the one hand, and the explanatory variables on the other hand (Figure 15). *Population density* shows the largest positive correlation with *reaction strength first wave* (0.60). The *finance sector* (0.55) and the *service sectors* (0.48) exhibit the second and third largest positive correlations, respectively. A possible explanation may be that the finance sector is dominated by white collar jobs which allow for remote work more easily. In contrast, the service sectors were completely shut down for large parts of the COVID-19 pandemic, forcing employees to stay home and consequently explaining the strong positive correlation. Finally, *green party* is also strongly positively correlated with *reaction strength first wave* (0.44). In contrast, employment sectors that do not allow any remote work and necessitate on-site labor, are strongly negatively correlated with *reaction strength first wave*: *agriculture, forestry, fisheries* (correlation coefficient of −0.47), *construction* (−0.44), and the *manufacturing sector* (−0.43). Examining the correlation coefficients among the explanatory variables reveals an extremely positive correlation between *average age* and *65+ year olds* (0.97). *Manufacturing sector* and *service sectors* are strongly negatively correlated (−0.99), and so are *voter turnout* and *unemployment rate* (−0.74). We further observe a strong negative correlation between *green party* and the right-wing party *AfD* (−0.70).

Examining *reaction strength second wave* yields similar patterns, though with slightly smaller correlation coefficients. Again, *reaction strength second wave* is strongly positively correlated with *population density* (0.50), the *finance sector* (0.48), *green party* (0.47), and the *service sectors* (0.46). Again, we observe strong negative correlations with *construction* (−0.44), the *manufacturing sector* (−0.43), *agriculture, forestry, fisheries* (−0.36).

Overall, the correlation analysis reveals that reaction strength in both waves is consistently positively correlated to population density, sectors amenable to remote work or disrupted by lockdowns, and green party support, while being inversely related to industries requiring on-site labor.

#### 4.5.2 Exhaustive search

We perform an exhaustive search of all possible variable combinations to determine which independent variables predict *reaction strength first wave* and *reaction strength second wave*. For this analysis step, we use the R package olsrr (50). For each subset size (1–20 variables), we select the model that maximized adjusted *R*^2^ (for first wave analysis, see Table 6 for variables chosen for each variable count and for second wave analysis, see Table 7). For the first wave, via this method, *population density*, the explanatory variable most strongly correlated with *reaction strength first wave*, is chosen for every subset size, while *unemployment rate*, the explanatory variable second-most-strongly correlated with *reaction strength first wave*, is chosen from a subset size of 5 onward. Overall, apart from population density, the variables chosen in the smaller models, mostly relate to different job sector and the necessity (or lack thereof) of on-site work: A high *unemployment rate*, working in the *finance sector* as well as having a higher *income* correlates with pursuing a white collar job, allowing for home office and remote work more readily. Contrarily, working in *agriculture, forestry, and fisheries* forces employees to work on-site. For the second wave, via this method, political variables gained importance, with at least one political party variable select from the two variable model onward. Conversely, *population density* is only chosen in the one variable model, the ten variables model, and from the twelve variables model onward.

**Table 6:** Regression on reaction strength of *first wave* favors population strength and demographic variables.

No. of variables	Variables chosen
1	Population density
2	Population density, Finance sector
3	Population density, Agriculture, forestry, fisheries, Finance sector
4	Population density, Agriculture, forestry, fisheries, Manufacturing sector, Service sector
5	Population density, Voter turnout, Unemployment rate, Agriculture, forestry, fisheries, Finance sector
6	Population density, Income, Unemployment rate, Agriculture, forestry, fisheries, Manufacturing Sector, Service sectors
7	Population density, Voter turnout, Income, Unemployment rate, Agriculture, forestry, fisheries, Manufacturing sector, Service sector
8	Population density, Voter turnout, Income, Small children in childcare, Unemployment rate, Agriculture, forestry, fisheries, Manufacturing sector, Service sectors
9	Population density, Voter turnout, Income, Small children in childcare, Green party, Unemployment rate, Agriculture, forestry, fisheries, Manufacturing sector, Service sector
10	Population density, Voter turnout, Income, Small children in childcare, Green party, Unemployment rate, Agriculture, forestry, fisheries, Manufacturing sector, Construction, Service sector
11	Population density, Voter turnout, Income, Small children in childcare, Green party, FDP, Unemployment rate, Agriculture, forestry, fisheries, Manufacturing sector, Construction, Service sector
12	Population density, Voter turnout, Income, Small children in childcare, SPD, Green party, AfD, Unemployment rate, Agriculture, forestry, fisheries, Manufacturing sector, Construction, Service sector
13	Population density, Voter turnout, Income, CDU, SPD, Green party, FDP, AfD, Unemployment rate, Agriculture, forestry, fisheries, Manufacturing sector, Construction, Service sector
14	Population density, Voter turnout, Income, Small children in childcare, CDU, SPD, Green party, FDP, AfD, Left party, Unemployment rate, Agriculture, forestry, fisheries, Manufacturing sector, Service sector
15	Population density, Voter turnout, Income, Small children in childcare, CDU, SPD, Green party, FDP, AfD, Left Party, Unemployment rate, Agriculture, forestry, fisheries, Manufacturing sector, Construction, Service sector
16	Population density, Voter turnout, Income, Small children in childcare, CDU, SPD, Green party, FDP, AfD, Left Party, Unemployment rate, Agriculture, forestry, fisheries, Manufacturing sector, Construction, Service sector, Finance sector
17	Population density, Voter turnout, Income, Small children in childcare, CDU, SPD, Green party, FDP, AfD, Left Party, Unemployment rate, Agriculture, forestry, fisheries, Manufacturing sector, Construction, Service sector, TTHIC sectors, Finance sector
18	Population density, Voter turnout, Income, Small children in childcare, Average age, CDU, SPD, Green party, FDP, AfD, Left Party, Unemployment rate, Agriculture, forestry, fisheries, Manufacturing sector, Construction, Service sector, TTHIC sectors, Finance sector
19	Population density, Voter turnout, Income, Small children in childcare, Average age, 65+ year olds, CDU, SPD, Green party, FDP, AfD, Left Party, Unemployment rate, Agriculture, forestry, fisheries, Manufacturing sector, Construction, Service sector, TTHIC sectors, Finance sector
20	Population density, Voter turnout, Income, Small children in childcare, Average age, 65+ year olds, CDU, SPD, Green party, FDP, AfD, Left Party, Employment rate, Unemployment rate, Agriculture, forestry, fisheries, Manufacturing sector, Construction, Service sector, TTHIC sectors, Finance sector

For each subset size (1–20 variables), all possible combinations are considered via an exhaustive search and the subset with the maximal adjusted *R*^2^ is selected.

**Table 7:** Regression on reaction strength of *second wave* favors political variables.

No. of Variables	Variables chosen
1	Population density
2	Population density, Service sector
3	Green party, Unemployment rate, Finance sector
4	Green party, FDP, Unemployment rate, Finance sector
5	Population density, Small children in childcare, AfD, 65+ year olds, Average age
6	Population density, Small children in childcare, FDP, AfD, 65+ year olds, Average age
7	Population density, Small children in childcare, FDP, AfD, 65+ year olds, Average age, Finance sector
8	Population density, Income, Small children in childcare, FDP, AfD, 65+ year olds, Average age, Unemployment rate
9	Population density, Income, Small children in childcare, SPD, FDP, AfD, 65+ year olds, Average age, Unemployment rate
10	Population density, Income, Small children in childcare, SPD, FDP, AfD, 65+ year olds, Average age, Unemployment rate, Agriculture, forestry, fisheries
11	Population density, Voter turnout, Income, Small children in childcare, SPD, FDP, AfD, 65+ year olds, Average age, Unemployment rate, Agriculture, forestry, fisheries
12	Population density, Income, Small children in childcare, SPD, FDP, AfD, 65+ year olds, Average age, Unemployment rate, Agriculture, forestry, fisheries, Manufacturing sector, Service sector
13	Population density, Voter turnout, Income, Small children in childcare, SPD, FDP, AfD, 65+ year olds, Average age, Unemployment rate, Agriculture, forestry, fisheries, Manufacturing sector, Service sector
14	Population density, Voter turnout, Income, Small children in childcare, SPD, FDP, AfD, 65+ year olds, Average age, Unemployment rate, Agriculture, forestry, fisheries, Manufacturing sector, Construction, Service sector
15	Population density, Voter turnout, Income, Small children in childcare, SPD, FDP, AfD, 65+ year olds, Average age, Unemployment rate, Agriculture, forestry, fisheries, Manufacturing sector, Construction, Service sector, Finance sector
16	Population density, Voter turnout, Income, Small children in childcare, SPD, FDP, AfD, Left party, 65+ year olds, Average age, Unemployment rate, Agriculture, forestry, fisheries, Manufacturing sector, Construction, Service sector, Finance sector
17	Population density, Voter turnout, Income, Small children in childcare, SPD, FDP, AfD, Left party, 65+ year olds, Average age, Unemployment rate, Agriculture, forestry, fisheries, Manufacturing sector, Construction, Service sector, TTHIC sectors, Finance sector
18	Population density, Voter turnout, Income, Small children in childcare, SPD, FDP, AfD, Left party, 65+ year olds, Average age, Unemployment rate, Employment rate, Agriculture, forestry, fisheries, Manufacturing sector, Construction, Service sector, TTHIC sectors Finance sector
19	Population density, Voter turnout, Income, Small children in childcare, CDU, SPD, FDP, AfD, Left party, 65+ year olds, Average age, Unemployment rate, Employment rate, Agriculture, forestry, fisheries, Manufacturing sector, Construction, Service sector, TTHIC sectors, Finance sector
20	Population density, Voter turnout, Income, Small children in childcare, CDU, SPD, Green party, FDP, AfD, Left party, 65+ year olds, Average age, Unemployment rate, Employment rate, Agriculture, forestry, fisheries, Manufacturing sector, Construction, Service sector, TTHIC sectors, Finance sector

For each subset size from 1 to 20 variables, all possible combinations are evaluated through exhaustive search, and the subset with the highest adjusted *R*^2^ is selected.

#### 4.5.3 Model selection

**Figure 16:**
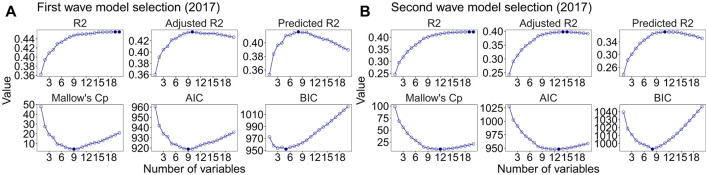
Model selection criteria for the two regressions favor the nine (first wave) and 12–13 (second wave) variable models. AIC is short for Akaike information criterion, BIC is short for Schwarz Bayesian information criterion. BIC favoring smaller models for both regressions is to be expected as BIC penalizes each additional parameter more strictly than AIC. **(A)** Model selection criteria for *reaction strength first wave* as regressand. Mallow's *C*_*p*_, and AICfavor the nine variable model, while adj. *R*^2^ favors the 10 variables and pred. *R*^2^ favors the eight variables model (marked with filled out circle). For each number of variables, the variables chosen through exhaustive search (Table 6) are considered. **(B)** Model selection criteria for *reaction strength second wave* as regressand. Apart from BIC, the model selection criteria favor the models with at least 12 variabless (marked with filled out circle). For each number of variables, the variables chosen through exhaustive search (Table 7) are considered.

We compare the following metrics across different subset sizes: *R*^2^, adjusted *R*^2^, pred *R*^2^, Mallow's C_p_, Akaike information criterion (AIC), and Schwarz Bayesian criterion (BIC). For the first wave, the majority of selection criteria favor the nine variable model, while for the second wave, the majority of selection criteria favor models with twelve or more variables (Figure 16). From here on forward, we consider the union of variables chosen this way: *population density, voter turnout, income, small children in childcare, SPD, Green party, FDP, AfD, average age, unemployment rate, agriculture, forestry, and fisheries, manufacturing sector*. The two variables not considered in the union but favored by the model selection criteria applied to the second wave are *65+ year olds* and *service sectors*. We exclude *65+ year olds* as it strongly positively correlates with *average age* and as we deem them to have the same effect on *reaction strength*. For the same reasoning we also excluded *service sectors* as it strongly negatively correlates with *manufacturing sector*.

#### 4.5.4 Regression on reaction strength

We regress *reaction strength first wave* and *reaction strength second wave* on the union of variables chosen via the model selection of the previous paragraph. Before interpreting the regression coefficients, we assess multicollinearity among predictors using variance inflation factors (VIF, see Methods subsection “Regression on reaction strength”). All variables except *unemployment rate* have VIFs below 5, indicating only modest multicollinearity. The VIF of *unemployment rate* (5.80) is slightly above this threshold, suggesting moderate multicollinearity with other predictors, most plausibly *income* and *voter turnout*. However, as the coefficient of *unemployment rate* is stable in sign and significance across both waves (see Methods subsection “Regression on reaction strength”), we retain it in the model and interpret its effect with appropriate caution. During the first COVID-19 wave, *population density* and *unemployment rate* contribute most strongly and equally to explaining variance in *reaction strength* (see Methods subsection “Regression on reaction strength”). *Voter turnout* and *income* are the other two significant (*p* < 0.05, not multiple comparison corrected) explanatory variable with a positive standardized coefficient. In contrast, *agriculture, forestry, fisheries* contributes to explaining variance in *reaction strength*, alas with a negative standardized coefficient (*p* < 0.001, not multiple comparison corrected).

During the second COVID-19 wave, *population density, unemployment rate*, and *agriculture, forestry, fisheries* remain statistically significant and their standardized regression coefficients have the same sign as during the first COVID-19 wave. Now, however, the election variables *FDP* and *AfD* are significant predictors (*p* < 0.001, not multiple comparison corrected), both with a negative correlation coefficient.

### 4.6 Structural equation modeling explaining cross-district differences in peak incidence

**Figure 17:**
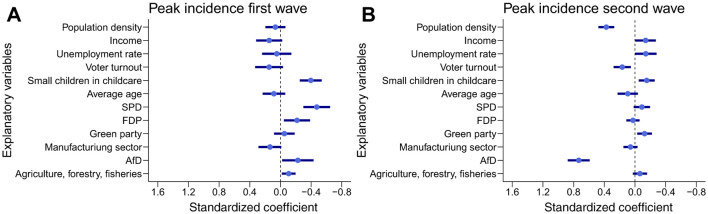
Total effects of demographic, socioeconomic, and political variables on *incidence*. Reaction strength serves as a mediator in the regression on incidence. Depiction of total effect size summing up direct (Figures 6C, D) and indirect (through reaction strength) effect of the socioeconomic variables on first wave **(A)** and second wave **(B)** incidence. In both panels, dots represent standardized coefficients, whiskers correspond to 95% confidence intervals.

To understand which factors impact *peak incidence* (here defined as the 90th percentile of the peak height of the corresponding wave), we employ a structural equation model using the R package lavaan (51). We assume that *reaction strength* as well as the twelve variables chosen via model selection (Section 4.5.3) impact peak incidence. However, as we have already shown that some of these twelve socioeconomic variables also impact reaction strength, reaction strength enters the structural equation model as a mediator. This allows the computation of the *total effect size* of the twelve economic variables, the sum of the direct effect size and the indirect effect size mediated through reaction strength (see Figure 17 for total effect sizes and Figure 6 for direct effect sizes).

### 4.7 Data sources

All data used in this work is publicly available (see Table 8 for details).

**Table 8:** Regression analysis is based solely on publicly available data from a variety of sources.

Data/Variable	Description	Source
Out-of home duration Temperature School vacations Public holidays COVID-19 case numbers Population density Income Unemployment rate Employment rate Average age 65+ year olds Small children in childcare Voter turnout CDU SPD Green party FDP AfD Left party Agriculture, forestry, fisheries Manufacturing sector Construction Service sectors TTHIC sectors Finance sector	Based on cell-based mobility data. For each zip code, the out- of-home duration sum over all recorded persons (in seconds) is available. Zip codes are grouped according to their district. For each district and each day, the out-of-home duration sum (in sec- onds) is computed, divided by the number of people living in this district and by 3600 to obtain the out-of-home duration (in hours) of an average person in the d. From the daily out-of-home durations, the weekly average is computed (see (16) for details) Weekly average of daily maximum temperature in (°*C*) for each German district. School vacations for each of the 16 federal states. Public holidays for each of the 16 federal states. COVID-19 7-Day Incidence/100.000 for each German district and all of Germany Extrapolation of inhabitants per km^2^ on December 31st, 2022 based on 2011 census Primary income of private households, including private non-profit organizations in 2020, in EURO Unemployment rate relative to the total civilian labor force (sum of working population and registered unemployed persons) in 2020, annual average, in % Division of the number of employees subject to social insurance contributions on June 30th, 2020 by the population aged 15-64 on December 31 of the previous year, in %. The 2020 *employment rate* was unavailable for the following districts: Suhl, Wartburgkreis, Schmalkalden-Meiningen, Ilm-Kreis, Sonneberg, Saalfeld-Rudolstadt. For these districts, we used the 2021 *employment rate* Average age of the population on December 31st, 2020 Percentage of people aged 65 and older in the total population in 2020 Share of children under the age of 3 in daycare facilities/daycare centers in the age group in 2020, in % Federal election turnout in 2017 in districts, in % Share of votes for political party *Christian Democratic Union of Germany* (CDU) in 2017 federal election, in % Share of votes for political party *Social Democratic Party of Germany* (SPD) in 2017 federal election, in % Share of votes for political party *Alliance 90/The Greens* (green party) in 2017 federal election, in % Share of votes for political party *Free Democratic Party* (FDP) in 2017 federal election , in % Share of votes for political party *Alternative for Germany* (AfD) in 2017 federal election, in % Share of votes for political party *The Left* in 2017 federal election, in % Proportion of employed persons in agriculture, forestry and fisheries in 2020, in % Proportion of employed persons in the manufacturing sector in 2020, in % Proportion of employed persons working in construction in 2020, in % Proportion of employed persons in service sectors in 2020, in % Proportion of employed persons in the trade, transport, hospitality, information, and communication sector in 2020, in % Proportion of employed persons in financial, insurance, and business services, real estate and housing sector in 2020, in %	(52) Meteostat (54) Schulferien.org (55) Schulferien.org (55) RKI (57) Statistisches Bundesamt (61) Statistische Ämter des Bundes und der Länder (62) Bundesagentur für Arbeit (56) Bundesagentur für Arbeit (60) Regionalatlas (63) Deutschlandatlas (59) Deutschlandatlas (58) GERDA: German Election Database (64) Regionalatlas (63) Regionalatlas (63) Regionalatlas (63) Regionalatlas (63) Regionalatlas (63) Regionalatlas (63) Regionalatlas (63) Regionalatlas (63) Regionalatlas (63) Regionalatlas (63) Regionalatlas (63) Regionalatlas (63)

All data was available for download at the time of writing.

## Data Availability

The original contributions presented in the study are included in the article/supplementary material, further inquiries can be directed to the corresponding author.

## References

[1] Perra N. Non-pharmaceutical interventions during the COVID-19 pandemic: a review. Phys Rep. (2021) 913:1–52. doi: 10.1016/j.physrep.2021.02.001 33612922 PMC7881715

[2] Santamaria C Sermi F Spyratos S Iacus SM Annunziato A Tarchi D . Measuring the impact of COVID-19 confinement measures on human mobility using mobile positioning data. A European regional analysis. Saf Sci. (2020) 132:104925. doi: 10.1016/j.ssci.2020.104925 32952303 PMC7486861

[3] Schlosser F Maier BF Jack O Hinrichs D Zachariae A Brockmann D. COVID-19 lockdown induces disease-mitigating structural changes in mobility networks. Proc Nat Acad Sci USA. (2020) 117:32883–90. doi: 10.1073/pnas.2012326117 33273120 PMC7776901

[4] Paltra S Bostanci I Nagel K. The effect of mobility reductions on infection growth is quadratic in many cases. Sci Rep. (2024) 14:14475. doi: 10.1038/s41598-024-64230-1 38914583 PMC11196635

[5] Lee KS Eom JK. Systematic literature review on impacts of COVID-19 pandemic and corresponding measures on mobility. Transportation. (2023) 51:1907–61. doi: 10.1007/s11116-023-10392-2 37363373 PMC10126540

[6] Müller SA Balmer M Charlton W Ewert R Neumann A Rakow C . Predicting the effects of COVID-19 related interventions in urban settings by combining activity-based modelling, agent-based simulation, and mobile phone data. PLoS ONE. (2021) 16:e0259037. doi: 10.1101/2021.02.27.21252583 34710158 PMC8553173

[7] Dönges P Wagner J Contreras S Iftekhar EN Bauer S Mohr SB . Interplay between risk perception, behavior, and COVID-19 spread. Front Phys. (2022) 10. doi: 10.3389/fphy.2022.842180

[8] Lim TY Xu R Ruktanonchai N Saucedo O Childs LM Jalali MS . Why similar policies resulted in different COVID-19 outcomes: how responsiveness and culture influenced mortality rates. Health Aff . (2023) 42:1637–46. doi: 10.1377/hlthaff.2023.00713 38048504

[9] Chan HF Skali A Savage DA Stadelmann D Torgler B. Risk attitudes and human mobility during the COVID-19 pandemic. Sci Rep. (2020) 10:19931. doi: 10.1038/s41598-020-76763-2 33199737 PMC7669857

[10] Nouvellet P Bhatia S Cori A Ainslie KEC Baguelin M Bhatt S . Reduction in mobility and COVID-19 transmission. Nat Commun. (2021) 12:1090. doi: 10.1038/s41467-021-21358-2 33597546 PMC7889876

[11] Lee KS Eom JK. Systematic literature review on impacts of COVID-19 pandemic and corresponding measures on mobility. Transportation. (2024) 51:1907–61. doi: 10.1007/s11116-023-10392-2 37363373 PMC10126540

[12] Li X Rudolph AE Mennis J. Association between population mobility reductions and new COVID-19 diagnoses in the United States along the urban-rural gradient, February-April, 2020. Prev Chronic Dis. (2020) 17:E118. doi: 10.5888/pcd17.200241 33006542 PMC7553217

[13] Natale F Iacus SM Conte A Spyratos S Sermi F. Territorial differences in the spread of COVID-19 in European regions and US counties. PLoS ONE. (2023) 18:e0280780. doi: 10.1371/journal.pone.0280780 36753502 PMC9907802

[14] Monnat SM. Rural-urban variation in COVID-19 experiences and impacts among US working-age adults. Ann Am Acad Pol Soc Sci. (2021) 698:111–36. doi: 10.1177/00027162211069717 35493266 PMC9055492

[15] Assche SB-V Ferraccioli F Riccetti N Gomez-Ramirez J Ghio D Stilianakis NI. Urban-rural disparities in COVID-19 hospitalisations and mortality: a population-based study on national surveillance data from Germany and Italy. PLoS ONE. (2024) 19:e0301325. doi: 10.1371/journal.pone.0301325 38696525 PMC11065260

[16] Mueller SA Paltra S Rehmann J Ewert R Nagel K. Comparing GPS and cell-based mobile phone data to identify activity participation during the COVID-19 pandemic. EPJ Data Sci. (2024) 13:71. doi: 10.1140/epjds/s13688-024-00510-0

[17] Anke J Francke A Schaefer LM Petzoldt T. Impact of SARS-CoV-2 on the mobility behaviour in Germany. Eur Trans Res Rev. (2021) 13:10. doi: 10.1186/s12544-021-00469-3 38624595 PMC7835317

[18] Järv O Ahas R Witlox F. Understanding monthly variability in human activity spaces: a twelve-month study using mobile phone call detail records. Transp Res Part C Emerg Technol. (2014) 38:122–35. doi: 10.1016/j.trc.2013.11.003

[19] Schönfelder S Axhausen KW. Urban Rhythms and Travel Behaviour. London: Routledge (2016). doi: 10.4324/9781315548715

[20] Lai S Sorichetta A Steele J Ruktanonchai CW Cunningham AD Rogers G . Global holiday datasets for understanding seasonal human mobility and population dynamics. Sci Data. (2022) 9:17. doi: 10.1038/s41597-022-01120-z 35058466 PMC8776767

[21] Cools M Moons E Wets G. Investigating effect of holidays on daily traffic counts: time series approach. Transp Res Rec. (2007) 2019:22–31. doi: 10.3141/2019-04

[22] Wheeler CC Erhart LM Jehn ML. Effect of school closure on the incidence of influenza among school-age children in Arizona. Public Health Rep. (2010) 125:851–9. doi: 10.1177/003335491012500612 21121230 PMC2966666

[23] Böhme S Burkert C Carstensen J Eigenhüller L Niebuhr A Roth D . Die Bedeutung der regionalen Wirtschaftsstruktur für die Arbeitsmarkteffekte der Corona-Pandemie: Eine erste Einschätzung. Nürnberg: IAB-Forschungsbericht (2020).

[24] Brodeur A Brodeur A Grigoryeva I Kattan L. Stay-at-Home Orders, Social Distancing and Trust. IZA Discussion Paper No. 13234. Available online at: https://ssrn.com/abstract=3602410

[25] Shanka MS Menebo MM. When and how trust in government leads to compliance with COVID-19 precautionary measures. J Bus Res. (2022) 139:1275–83. doi: 10.1016/j.jbusres.2021.10.036 34744211 PMC8559780

[26] Dingel JI Neiman B. How many jobs can be done at home? J Public Econ. (2020) 189:104235. doi: 10.1016/j.jpubeco.2020.104235 32834177 PMC7346841

[27] Badr HS Du H Marshall M Dong E Squire MM Gardner LM. Association between mobility patterns and COVID-19 transmission in the USA: a mathematical modelling study. Lancet Infect Dis. (2020) 20:1247–54. doi: 10.1016/S1473-3099(20)30553-3 32621869 PMC7329287

[28] Alirol E Getaz L Stoll B Chappuis F Loutan L. Urbanisation and infectious diseases in a globalised world. Lancet Infect Dis. (2011) 11:131–41. doi: 10.1016/S1473-3099(10)70223-1 21272793 PMC7106397

[29] Teller J. Urban density and COVID-19: towards an adaptive approach. Build Cities. (2021) 2:150–65. doi: 10.5334/bc.89

[30] Murray T. Stay-at-home orders, mobility patterns, and spread of COVID-19. Am J Public Health. (2021) 111:1149–56. doi: 10.2105/AJPH.2021.306209 33856875 PMC8101571

[31] Yan Y Malik AA Bayham J Fenichel EP Couzens C Omer SB. Measuring voluntary and policy-induced social distancing behavior during the COVID-19 pandemic. Proc Nat Acad Sci USA. (2021) 118:e2008814118. doi: 10.1073/pnas.2008814118 33820846 PMC8076999

[32] Summan A Nandi A. Timing of non-pharmaceutical interventions to mitigate COVID-19 transmission and their effects on mobility: a cross-country analysis. Eur J Health Econ. (2021) 23:105–17. doi: 10.1007/s10198-021-01355-4 34304325 PMC8310614

[33] Petherick A Goldszmidt R Andrade EB Furst R Hale T Pott A . A worldwide assessment of changes in adherence to COVID-19 protective behaviours and hypothesized pandemic fatigue. Nat Human Behav. (2021) 5:1145–60. doi: 10.1038/s41562-021-01181-x 34345009

[34] World Health Organization Regional Office for Europe. Pandemic Fatigue-Reinvigorating the Public to Prevent COVID-19: Policy Framework for Supporting Pandemic Prevention and Management. Copenhagen: World Health Organization. Regional Office for Europe (2020). Available online at: https://www.who.int/europe/publications/i/item/WHO-EURO-2020-1573-41324-56242 (Accessed June 24, 2026).

[35] Hale T Angrist N Goldszmidt R Kira B Petherick A Phillips T . A global panel database of pandemic policies (Oxford COVID-19 Government Response Tracker). Nat Human Behav. (2021) 5:529–38. doi: 10.1038/s41562-021-01079-8 33686204

[36] Wesolowski A Buckee CO Engø-Monsen K Metcalf CJE. Connecting mobility to infectious diseases: the promise and limits of mobile phone data. J Infect Dis. (2016) 214:S414–20. doi: 10.1093/infdis/jiw273 28830104 PMC5144902

[37] Grantz KH Meredith HR Cummings DAT Metcalf CJE Grenfell BT Giles JR . The use of mobile phone data to inform analysis of COVID-19 pandemic epidemiology. Nat Commun. (2020) 11:4961. doi: 10.1038/s41467-020-18190-5 32999287 PMC7528106

[38] Oliver N Lepri B Sterly H Lambiotte R Deletaille S De Nadai M . Mobile phone data for informing public health actions across the COVID-19 pandemic life cycle. Sci Adv. (2020) 6:eabc0764. doi: 10.1126/sciadv.abc0764 32548274 PMC7274807

[39] Buckee CO Balsari S Chan J Crosas M Dominici F Gasser U . Aggregated mobility data could help fight COVID-19. Science. (2020) 368:145–6. doi: 10.1126/science.abb8021 32205458

[40] Chang S Pierson E Koh PW Gerardin J Redbird B Grusky D . Mobility network models of COVID-19 explain inequities and inform reopening. Nature. (2021) 589:82–7. doi: 10.1038/s41586-020-2923-3 33171481

[41] Rader B White LF Burns MR Chen J Brilliant J Cohen J . Crowding and the shape of COVID-19 epidemics. Nat Med. (2020) 26:1829–34. doi: 10.1038/s41591-020-1104-0 33020651

[42] Madewell ZJ Yang Y Longini IM Halloran ME Dean NE. Household transmission of SARS-CoV-2: a systematic review and meta-analysis. JAMA Netw Open. (2020) 3:e2031756. doi: 10.1001/jamanetworkopen.2020.31756 33315116 PMC7737089

[43] Statista Research Department. Germany: Smartphone Penetration rate 2020-2029. Statista – statista.com. Available online at: https://www.statista.com/statistics/568095/predicted-smartphone-user-penetration-rate-in-germany/ (Accessed May 22, 2026).

[44] Docquier F Golenvaux N Nijssen S Schaus P Stips F. Cross-border mobility responses to COVID-19 in Europe: new evidence from Facebook data. Global Health. (2022) 18:41. doi: 10.1186/s12992-022-00832-6 35436927 PMC9013976

[45] Goolsbee A Syverson C. Fear, lockdown, and diversion: comparing drivers of pandemic economic decline 2020. J Public Econ. (2021) 193:104311. doi: 10.1016/j.jpubeco.2020.104311 33262548 PMC7687454

[46] Henning-Smith C Kozhimannil K Evenson A. Addressing Commuting as a Public Health Issue: Strategies Should Differ by Rurality. Minneapolis, MN: University of Minnesota Rural Health Research Center (2018). p. 1–5.

[47] Abril-Pla O Andreani V Carroll C Dong L Fonnesbeck CJ Kochurov M . PyMC: a modern, and comprehensive probabilistic programming framework in Python. PeerJ Comput Sci. (2023) 9:e1516. doi: 10.7717/peerj-cs.1516 37705656 PMC10495961

[48] Hoffman MD Gelman A. The No-U-Turn sampler: adaptively setting path lengths in Hamiltonian Monte Carlo. J Mach Learn Res. (2014) 15:1593–623.

[49] Seyboldt A. nutpie: A Fast Sampler for Bayesian Posteriors (2024). Python package version 0.15.2. Available online at: https://github.com/pymc-devs/nutpie (Accessed June 24, 2026).

[50] Hebbali A. olsrr: Tools for Building OLS Regression Models (2024). R package version 0.6.1.9000. Available online at: https://github.com/rsquaredacademy/olsrr (Accessed June 24, 2026).

[51] Rosseel Y. lavaan: an R package for structural equation modeling. J Stat Softw. (2012) 48:1–36. doi: 10.18637/jss.v048.i02

[52] Balmer M Ewert R Müller SA Nagel K Neumann A Rakow C. Mobility data on county level (Version 1) [Data set]. Cluster of Excellence MATH+ (2022). doi: 10.5281/zenodo.6602614

[53] an der Heiden M. SARS-CoV-2-Nowcasting und -R-Schaetzung. Zenodo (2023).

[54] Meteostat. Meteostat Bulk Data (2023). Available online at: https://dev.meteostat.net/bulk/ (Accessed June 24, 2026).

[55] Schulferien.org. Ferien, Feiertage, Kalender. Available online at: https://www.schulferien.org/ (Accessed April 16, 2025).

[56] Bundesagentur für Arbeit. Arbeitslose und Arbeitslosenquoten - Deutschland, Länder, Kreise und Gemeinden (Zeitreihe Monats- und Jahreszahlen) (2020). Available online at: https://statistik.arbeitsagentur.de/SiteGlobals/Forms/Suche/Einzelheftsuche_Formular.html?nn=1610104&topic_f=gemeinde-arbeitslose-quoten (Accessed October 23, 2025).

[57] Robert Koch-Institut. 7-Tage-Inzidenz der COVID-19-Fälle in Deutschland (2025). Available online at: https://robert-koch-institut.github.io/COVID-19_7-Tage-Inzidenz_in_Deutschland/ (Accessed June 24, 2026).

[58] Deutschlandatlas. Datenbasis: Laufende Raumbeobachtung des BBSR; Statistik der öffentlich geförderten Kindertagespflege; Regionaldatenbank der Statistischen Ämter des Bundes und der Länder. Anteil der betreuten Kinder unter 3 Jahren in Kindertageseinrichtungen/-tagespflege an der Altersgruppe im Jahr 2020 in % (2025). Available online at: https://www.deutschlandatlas.bund.de/SharedDocs/Downloads/DE/HA20/Deutschlandatlas_KRS1218_HA20.html (Accessed September 15, 2025).

[59] Deutschlandatlas. Datenbasis: Laufende Raumbeobachtung des BBSR; Fortschreibung des Bevölkerungsstandes, Regionaldatenbank der Statistischen Ämter des Bundes und der Länder. Anteil der 65-Jährigen und Älteren an der Gesamtbevölkerung im Jahr 2020 in % (2020). Available online at: https://www.deutschlandatlas.bund.de/SharedDocs/Downloads/DE/HA20/Deutschlandatlas_KRS1218_HA20.html (Accessed September 18, 2025).

[60] Bundesagentur für Arbeit. Beschäftigungsquoten (SvB, GB, aGB) - Deutschland, Länder, Kreise und Agenturen für Arbeit (Jahreszahlen und Zeitreihen) (2020). Available online at: https://statistik.arbeitsagentur.de/SiteGlobals/Forms/Suche/Einzelheftsuche_Formular.html?gtp=15084_list%253D2&topic_f=beschaeftigung-sozbe-bq-heft (Accessed October 23, 2025)

[61] Bundesamt DS. Kreisfreie Städte und Landkreise nach Fläche, Bevölkerung und Bevölkerungsdichte am (2024). Available online at: https://www.destatis.de/DE/Themen/Laender-Regionen/Regionales/Gemeindeverzeichnis/Administrativ/04-kreise.html (Accessed September 15, 2025).

[62] Statistische Ämter des Bundes und der Länder. Einkommen der privaten Haushalte in den kreisfreien Städten und Landkreisen der Bundesrepublik Deutschland (2023). Available online at: https://www.statistikportal.de/de/vgrdl/ergebnisse-kreisebene/einkommen-kreise (Accessed September 15, 2025).

[63] Statistische Ämter des Bundes und der Länder. Regionalatlas Deutschland | Kartenanwendung – regionalatlas.*statistikportal.de* (2020). Available online at: https://regionalatlas.statistikportal.de/ (Accessed September 20, 2025).

[64] Heddesheimer V Hilbig H Sichart F Wiedemann A. GERDA: The German Election Database. Sci Data. (2025) 12:618. doi: 10.1038/s41597-025-04811-5 40229253 PMC11997170

