# Supplementary material for: Inferring mobility reductions from COVID-19 disease spread along the urban-rural gradient
Source: Front Public Health. 2026 Jul 6;14:1840916. doi: 10.3389/fpubh.2026.1840916 (PMC13381793; doi:10.3389/fpubh.2026.1840916)
Supplement: Supplementary file 1 [file Supplementary_file_1.pdf]

Supplementary Material

Comparison of different mobility measures

We use *out-of-home duration* as the outcome variable in our Bayesian hierarchical model. We also considered two alternative mobility metrics, distance traveled per person (km) and share of mobile persons, but found both to be strongly correlated with out-of-home duration (Fig. 1), offering no analytical advantage as substitutes.

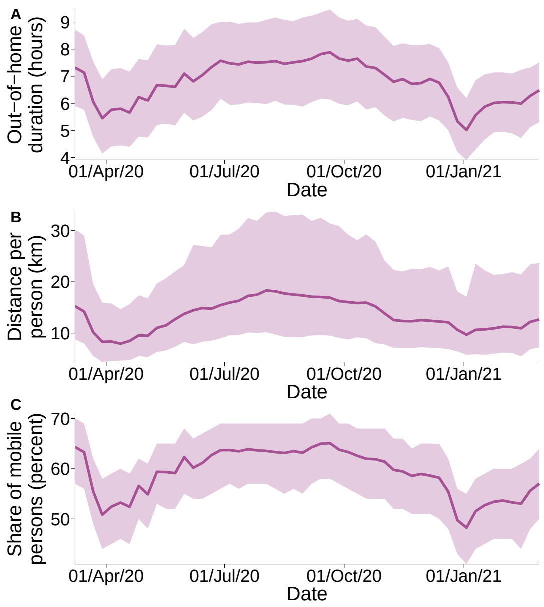

Figure 1: **Across districts, all three mobility measures show a sharp decline in spring 2020, values that exceed pre-pandemic levels in summer 2020, and a further decline in winter 2020/2021.** *Out-of-home duration* and *distance per person* are strongly correlated (Pearson's correlation coefficient = 0.92), as are *out-of-home duration* and *share of mobile persons* (Pearson's correlation coefficient = 0.99).

Effect sizes of temperature, school vacations, and public holidays in non-pandemic years

To test whether disease spread in our 2020 model masks the contributions of temperature, public holidays, and school vacations to out-of-home duration, we conducted a sensitivity analysis by fitting our Bayesian hierarchical model to 2024 data. Although COVID-19 cases were still reported in Germany in 2024, all NPIs had been discontinued by then. We therefore assume that disease spread no longer affected out-of-home duration, and accordingly omit it from the 2024 model, isolating the effects of temperature, school vacations, and public holidays:

$$D_d(t) = D_{\text{base},d} \cdot W_d(t) \cdot V_d(t) \cdot H_d(t).$$

The 2024 results are consistent with those of 2020: the influence of all three factors is comparable across districts, and out-of-home duration remains approximately 10% higher in summer than in winter due to temperature fluctuations (Fig. 2). Effect sizes for temperature and school vacations are similar across both years, while public holidays show a slightly larger influence in 2024. This correspondence suggests that disease spread did not substantially mask the contributions of these factors in our 2020 model.

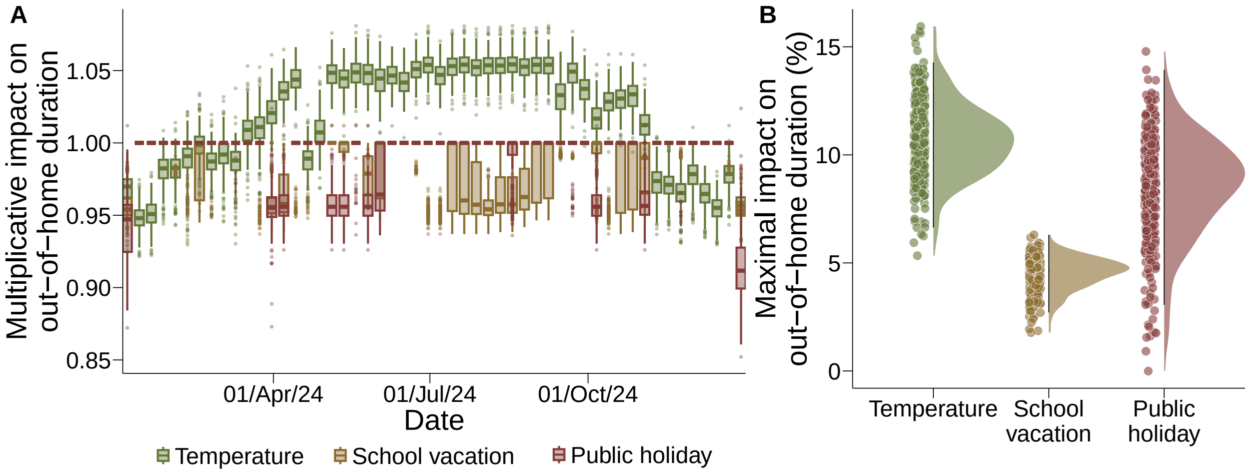

Figure 2: **Summary of the Bayesian models results for 2024. A. The influence of the different factors is comparable across districts and similar to our 2020 results.** As in 2020, due to temperature fluctuations, out-of-home duration is approximately 10% higher in summer than in winter. **B. Effect sizes of temperature and school vacations for 2024 are comparable to those of 2020, while influence of public holidays is slightly larger.** Distribution of 2024 effect sizes, in other words size of impact on out-of-home duration, across districts, using the median effect size for each district.

Alternative functional forms for weather effects and model comparison

In our Bayesian hierarchical model, we model the effect of temperature on out-of-home duration as increasing during the warmer months and decreasing during the colder months, using a sigmoid function. This imposes the assumption that no matter how hot it gets, temperature never decreases the out-of-home duration. In countries that experience extreme temperatures and potentially high humidity that drive people into the air-conditioned indoors, this is unlikely. To test, if we can also observe a decreasing effect of temperature in Germany, we modeled the temperature factor using a second and a fourth order polynomial. Using a second

order polynomial, the temperature factor reads:

$$\begin{aligned}
W_{d,quadratic}(t) &= -\phi_{W,d} \cdot (t_{\max,d}(t) + \psi_{W,d})^2 + \chi_{W,d},\\
\phi_{W,d} &= \mu_W^{\phi} + \sigma_W^{\phi} \cdot z_{W,d}^{\phi},\\
z_{W,d}^{\phi} &\sim \mathcal{N}(0,1),\\
\mu_W^{\phi} &\sim \mathcal{N}(0.001, 0.0001),\\
\sigma_W^{\phi} &\sim \mathrm{Exp}(10),\\
\psi_{W,d} &= \mu_W^{\psi} + \sigma_W^{\psi} \cdot z_{W,d}^{\psi},\\
z_{W,d}^{\psi} &\sim \mathcal{N}(0,1),\\
\mu_W^{\psi} &\sim \mathcal{N}(-20, 0.5),\\
\sigma_W^{\psi} &\sim \mathrm{Exp}(10),\\
\chi_{W,d} &= \mu_W^{\chi} + \sigma_W^{\chi} \cdot z_{W,d}^{\chi},\\
z_{W,d}^{\chi} &\sim \mathcal{N}(0,1),\\
\mu_W^{\chi} &\sim \mathcal{N}(1, 0.05),\\
\sigma_W^{\chi} &\sim \mathrm{Exp}(10).
\end{aligned}$$

Using a fourth order polynomial, the temperature factor reads:

$$\begin{aligned}
W_{d,quartic}(t) &= -\phi_{W,d} \cdot (t_{\max,d}(t) + \psi_{W,d})^4 + \chi_{W,d},\\
\phi_{W,d} &= \mu_W^{\phi} + \sigma_W^{\phi} \cdot z_{W,d}^{\phi},\\
z_{W,d}^{\phi} &\sim \mathcal{N}(0,1),\\
\mu_W^{\phi} &\sim \mathcal{N}(0.00001, 0.00001),\\
\sigma_W^{\phi} &\sim \mathrm{Exp}(10),\\
\psi_{W,d} &= \mu_W^{\psi} + \sigma_W^{\psi} \cdot z_{W,d}^{\psi},\\
z_{W,d}^{\psi} &\sim \mathcal{N}(0,1),\\
\mu_W^{\psi} &\sim \mathcal{N}(-20, 0.5),\\
\sigma_W^{\psi} &\sim \mathrm{Exp}(10),\\
\chi_{W,d} &= \mu_W^{\chi} + \sigma_W^{\chi} \cdot z_{W,d}^{\chi},\\
z_{W,d}^{\chi} &\sim \mathcal{N}(0,1),\\
\mu_W^{\chi} &\sim \mathcal{N}(1, 0.1),\\
\sigma_W^{\chi} &\sim \mathrm{Exp}(10).
\end{aligned}$$

Analogous to the main model, all free variables, the leading coefficient $\phi_{W,d}$, the horizontal shift $\psi_{W,d}$, and the offset $\chi_{W,d}$, are modeled hierarchically across districts. For these alternative model formulations, the disease factors $C_d(t)$, the school vacation factor $V_d(t)$, and the public holiday factor $H_d(t)$ are modeled analogously as in the main model. Only the temperature factor is replaced by the alternative formulations. Like for the main model, we perform Bayesian inference for the parameters of our model using Markov-Chain Monte Carlo sampling.

**Temperature factor**	**Rank**	**ELPD LOO**	**ELPD difference**	**ELPD difference standard error**	**Weight**
Sigmoidal	1	−9005.11	0	0	0.997
2nd order polynomial	2	−11802.08	2796.97	78.91	0.000
4th order polynomial	3	−19188.77	10183.66	285.22	0.003

Table 1: **The modal comparison favors the sigmoidal temperature factor.** Leave-one-out cross-validation, an estimate of the out-of-sample predictive fit, was used for model comparison. The weights provided in column five may loosely be interpreted as the probability of each model being true.

To compare model fits, we used leave-one-out cross-validation, which consistently favored the sigmoidal temperature factor (Table 1), supporting its inclusion in the main model.
